# Transcriptome of *Saccharomyces cerevisiae* during production of D-xylonate

**DOI:** 10.1186/1471-2164-15-763

**Published:** 2014-09-05

**Authors:** Dominik Mojzita, Merja Oja, Eija Rintala, Marilyn Wiebe, Merja Penttilä, Laura Ruohonen

**Affiliations:** VTT Technical Research Centre of Finland, P.O. Box 1000, Espoo, FI-02044 VTT Finland

**Keywords:** *Saccharomyces cerevisiae*, D-xylose, D-xylonate production, Microarrays, Stress response, Cell wall integrity pathway, Weak organic acids

## Abstract

**Background:**

Production of D-xylonate by the yeast *S. cerevisiae* provides an example of bioprocess development for sustainable production of value-added chemicals from cheap raw materials or side streams. Production of D-xylonate may lead to considerable intracellular accumulation of D-xylonate and to loss of viability during the production process. In order to understand the physiological responses associated with D-xylonate production, we performed transcriptome analyses during D-xylonate production by a robust recombinant strain of *S. cerevisiae* which produces up to 50 g/L D-xylonate.

**Results:**

Comparison of the transcriptomes of the D-xylonate producing and the control strain showed considerably higher expression of the genes controlled by the cell wall integrity (CWI) pathway and of some genes previously identified as up-regulated in response to other organic acids in the D-xylonate producing strain. Increased phosphorylation of Slt2 kinase in the D-xylonate producing strain also indicated that D-xylonate production caused stress to the cell wall. Surprisingly, genes encoding proteins involved in translation, ribosome structure and RNA metabolism, processes which are commonly down-regulated under conditions causing cellular stress, were up-regulated during D-xylonate production, compared to the control. The overall transcriptional responses were, therefore, very dissimilar to those previously reported as being associated with stress, including stress induced by organic acid treatment or production. Quantitative PCR analyses of selected genes supported the observations made in the transcriptomic analysis. In addition, consumption of ethanol was slower and the level of trehalose was lower in the D-xylonate producing strain, compared to the control.

**Conclusions:**

The production of organic acids has a major impact on the physiology of yeast cells, but the transcriptional responses to presence or production of different acids differs considerably, being much more diverse than responses to other stresses. D-Xylonate production apparently imposed considerable stress on the cell wall. Transcriptional data also indicated that activation of the PKA pathway occurred during D-xylonate production, leaving cells unable to adapt normally to stationary phase. This, together with intracellular acidification, probably contributes to cell death.

**Electronic supplementary material:**

The online version of this article (doi:10.1186/1471-2164-15-763) contains supplementary material, which is available to authorized users.

## Background

D-xylonate is an attractive platform chemical which can be produced from non-food carbohydrates by microbial conversion. D-xylonate can be used as a complexing agent or chelator, in dispersal of concrete and as a precursor for compounds such as co-polyamides, hydrogels and 1,2,4,-butanetriol [1–3]. In addition, it has potential as substitute for D-gluconate, which is widely used in pharmaceuticals, food products, solvents, adhesives, dyes, paints and polishes (reviewed in [4]).

D-xylonate is naturally produced by various bacteria, such as *Gluconobacter, Enterococcus, Enterobacter* and *Pseudomonas* species (reviewed in [5]). These organisms generally use membrane bound D-xylose or D-glucose oxidases for conversion of D-xylose to D-xylonate and high production of this acid has been achieved from pure D-xylose. However, when industrial hydrolysates would be used as substrates, the yield and purity of the product is expected to be compromised. High production of D-xylonate has also been demonstrated in genetically modified prokaryotic and eukaryotic micro-organisms [6–8]. The advantages of using eukaryotic micro-organisms, such as *Saccharomyces cerevisiae*, are good tolerance to inhibitors present in the hydrolysates and ability to produce acids in low pH. We have previously engineered *S. cerevisiae* strains for production of D-xylonate by expressing D-xylose dehydrogenases from *Caulobacter crescentus, Trichoderma reesei* and pig liver in this yeast [6, 9]. The highest production (43 g/L D-xylonate from 49 g/L D-xylose) of D-xylonate with *S. cerevisiae* was obtained with an industrial, hydrolysate-tolerant strain expressing an NAD^+^-dependent D-xylose dehydrogenase, XylB, from *C. crescentus*. However, the high production of D-xylonate in *S. cerevisiae* leads to dramatically decreased cell viability, especially in later stages of the production process [9].

Even though *S. cerevisiae* has good tolerance of weak organic acids, they cause stress. At least four different cellular systems have been proposed to be involved in the regulation of weak acid-induced stress in *S. cerevisiae*: 1) the general stress response pathway, regulated by transcription factors Msn2p/Msn4p [10], 2) the pathway specific for moderately lipophilic weak acids, mediated by transcription factor War1 [10, 11], 3) the pathway required for adaptation and resistance to the more hydrophilic acids (acetic and propionic), regulated by Haa1 [12–14], and 4) the RIM101 pathways, originally identified as being responsible for alkaline pH responses in yeast, but more recently shown to also be involved in responses to propionic acid [15]. In addition, the transcription factor Pdr1 has been shown to play a role in resistance to medium chain fatty acids [16], further emphasizing the complex nature of the weak acid stress response and its regulation in yeast.

No general "weak organic acid response” can be observed, based on the response of *S. cerevisiae* to diverse weak organic acids with different degrees of lipophilicity [17, 18]. Only one gene, *TPO3*, encoding a multidrug resistance transporter was upregulated in response to all organic acids tested in several studies which focused on weak organic acid treatment of yeast (reviewed in [17]). However, acids with similar properties, clearly induced overlapping responses, especially in transcription of genes related to cell wall metabolism (in the case of lipophilic acids), and genes encoding proteins associated with trans-membrane transport [18].

Here we present a transcriptional analysis of D-xylonate production by an industrial, hydrolysate tolerant *S. cerevisiae* [9] at different stages of the production process, in order to understand the physiological responses associated with D-xylonate production in *S. cerevisiae* and especially to uncover mechanisms leading to the loss of viability. These results are considered within the broader context of other published studies which have determined transcriptional responses of *S. cerevisiae* to weak acid stress, either from externally added, or, as with D-xylonate, from production of weak organic acids.

## Results

**Figure 1:**
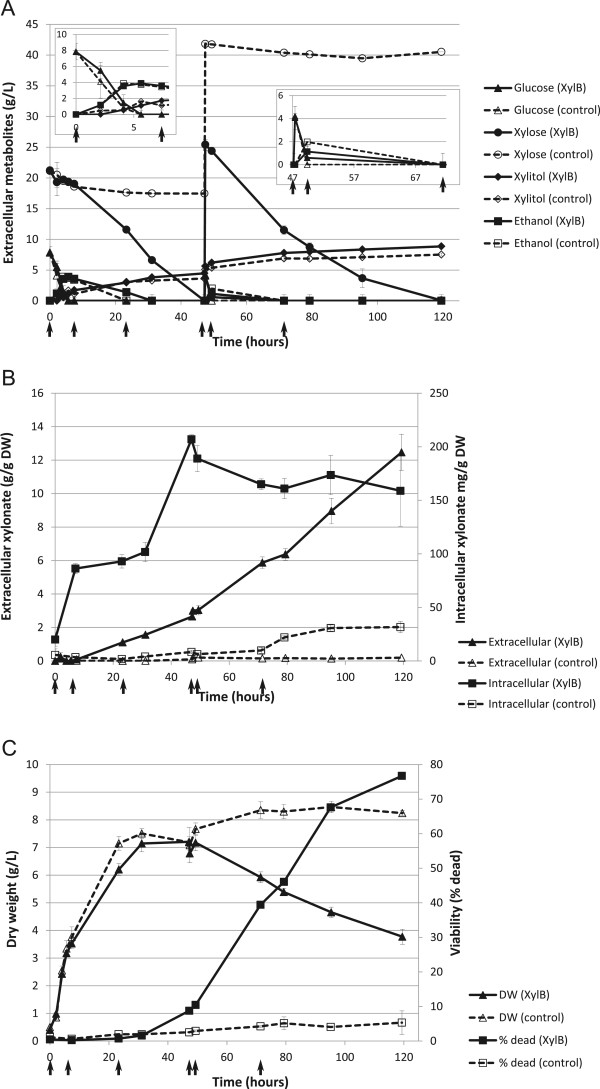
**Physiological data from bioreactor cultivations. A)** Extracellular concentrations of D-glucose, D-xylose, xylitol and ethanol. At 47 hours, 4 g/L D-glucose and 25 g/L D-xylose were added to the cultures. One insert shows the first 8 hours of cultivation, and another the hours after addition of D-glucose and D-xylose at 47 h. **B)** Extracellular and intracellular concentrations of D-xylonate. **C)** Biomass and cell viability. Arrows indicate the times when samples for microarrays were taken. The experiments were performed in triplicate; average ± standard deviation is shown.

D-Xylonate was produced by the industrial, hydrolysate tolerant *S. cerevisiae* strain B67002, expressing the *xylB* gene from *C. crescentus* [9]. The D-glucose consumption rate and the corresponding ethanol production rate were similar for the D-xylonate producing and the control strains, but ethanol consumption was slower for the D-xylonate producing strain (Figure 1A). Production of xylitol was comparable in the two strains, resulting from intrinsic D-xylose reductase activity, such as the aldose reductase, Gre3 [19].

Already at 7 hours, D-xylonate had accumulated considerably inside the *xylB*-expressing cells, to levels corresponding to about 10% of their dry biomass [9]. The concentration of D-xylonate continued to increase inside the cells until 47 hours (Figure 1B). Only small amounts (0.25 ± 0.14 g/L) of D-xylonate were observed in the culture supernatant at 7 hours, but the concentration increased continually until the end of the experiment at 120 hours. A decrease in cell viability of the *xylB*-expressing strain was detected at 47 hours and viability progressively decreased throughout the production process (Figure 1C, [9]). By 120 hours, 84 ± 16% of the D-xylonate producing cells were dead. In contrast, the control strain retained its viability throughout the experiment (Figure 1C, [9]).

Transcriptional profiling of the *xylB*-expressing, D-xylonate producing strain and the control strain focused on times of key physiological changes for the production strain (Figure 1A). Samples were taken immediately after inoculation (0 h); after D-glucose had been consumed, but with ethanol present, with measurable intracellular D-xylonate and very low extracellular D-xylonate (7 h), capturing early responses to D-xylonate production; and after ethanol had been consumed (by the control strain) and 7 ± 0.3 g/L extracellular D-xylonate being present, but viability remaining high (23 h), capturing intermediate responses. Samples at 47 h, after the D-xylonate producing strain had consumed the initial D-xylose, but before addition of more D-glucose and D-xylose, provided data for conditions with the highest intracellular D-xylonate concentration and measureable but low loss of viability in the production strain. The effect of addition of D-glucose, to provide energy and potentially improve D-xylonate export, was assessed at 49 h, 2 h after the addition when the D-glucose had already been consumed. The late phase of the production process and the stress responses associated with it were assessed with the 71 h sample, after significant loss of viability in the D-xylonate producing strain, but with still 48 ± 5% of the cells alive.

### Transcriptome analysis of D-xylonate producing cells and observations from gene clustering

The microarray data quality was confirmed by standard quality control methods (not shown) and the data was normalized using the RMA method. Two clustering approaches were used to visualize gene groups that had distinct transcriptional profiles. Both clustering methods used fuzzy c-means clustering [20]. The first approach highlighted the differences between the two strains and was based on fold changes (FC) of gene expression between the D-xylonate producing and the control strain with time. The second clustering approach was used to analyse the expression profiles with time, for each strain separately. In this second approach normalized profiles (NP) were used as input to the clustering algorithm to emphasize the “shape” of the expression profiles with time and to capture subtle differences in the up- or down-regulation of each gene, since these are not observed when absolute values or fold-change expression profiles are assessed.

**Figure 2:**
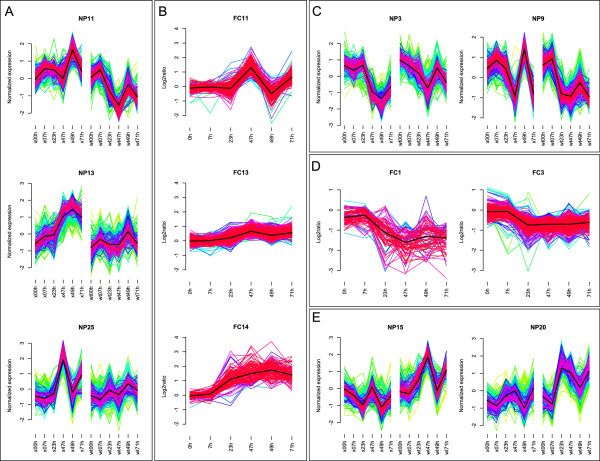
**Expression profiles of genes showing interesting differences between the strains. A, B)** Clusters containing genes that showed higher relative expression in the D-xylonate producing strain compared to the control strain: **A)** Clusters NP11, NP13 and NP25 identified in the normalized profiles clustering analysis, and **B)** corresponding clusters FC11, FC13 and FC14 from the fold-change clustering analysis. **C)** Clusters NP2 and NP3 from the normalized profile clustering, containing genes with delayed response (at 23 h) in the D-xylonate producing strain. **D, E)** Clusters containing genes showing relatively lower expression in the D-xylonate producing strain; **D)** Clusters FC1 and FC3 from the fold change clustering, and **E)** corresponding clusters NP15 and NP20 from the normalized profile clustering showing the relativity of the apparent down-regulation seen in clusters FC1 and FC3. The numbers of genes shared between NP and FC clusters are listed in Additional file 3.

Clustering resulted in 25 distinct NP clusters (Additional file 1) and 16 distinct FC clusters (Additional file 2). The most relevant set of clusters are shown in Figure 2. In order to identify genes showing expression changes dependent on D-xylonate production, our analysis focused on clusters with expression profiles consistent with the parameters observed during production of D-xylonate, such as the gradual accumulation of D-xylonate, dissimilar utilization of ethanol, and loss of viability. Therefore, we selected clusters which contained genes with either steady up- or down-regulation during D-xylonate production and did not carry out a detailed investigation of clusters with small or insignificant differences or with transient expression changes between the production and the control strains. Less emphasis was given to the sample at 49 hours, since the expression profiles of a vast number of genes were apparently influenced by the major physiological impact of D-glucose addition two hours earlier, which was not the focus of this study. Gene expression of many genes for the two strains did differ dramatically at this time, so many clusters contain genes with changed expression at this time only, but did not appear to be directly linked to the D-xylonate production.

Results from FC (strain differences) and NP (time effects) clustering methods were compared to determine how many genes each NP cluster had in common with each FC cluster (Additional file 3) and how genes were distributed in the individual clusters by each method. Genes which were observed in one NP cluster were usually present in one or two FC clusters. On the other hand, genes in one FC cluster were found in several NP clusters, since the observed change in FC can be achieved in several ways. For example, cluster FC13 (Figure 2B) contains genes that were increasingly upregulated during D-xylonate production. In NP clustering these genes belong mainly to clusters NP11 (Figure 2A, down-regulation in control strain over time) and NP13 (Figure 2A, up-regulation during D-xylonate production) that have different profiles compared to each other. Some genes in FC13 also fall into clusters NP2 and NP24 (Additional file 1), in which the shapes of the expression profiles in the two cultivations are similar, but the expression level is higher during D-xylonate production compared to the control (*i.e.* less down-regulated in NP2 and more up-regulated in NP24). Detailed information about the clustering is given in Additional file 4, in which the cluster memberships of all genes are listed together with the expression profiles (normalized expression profile and log2 ratio profile) used as the input to the clusterings.

### Groups of genes with increased expression during D-xylonate production

**Figure 3:**
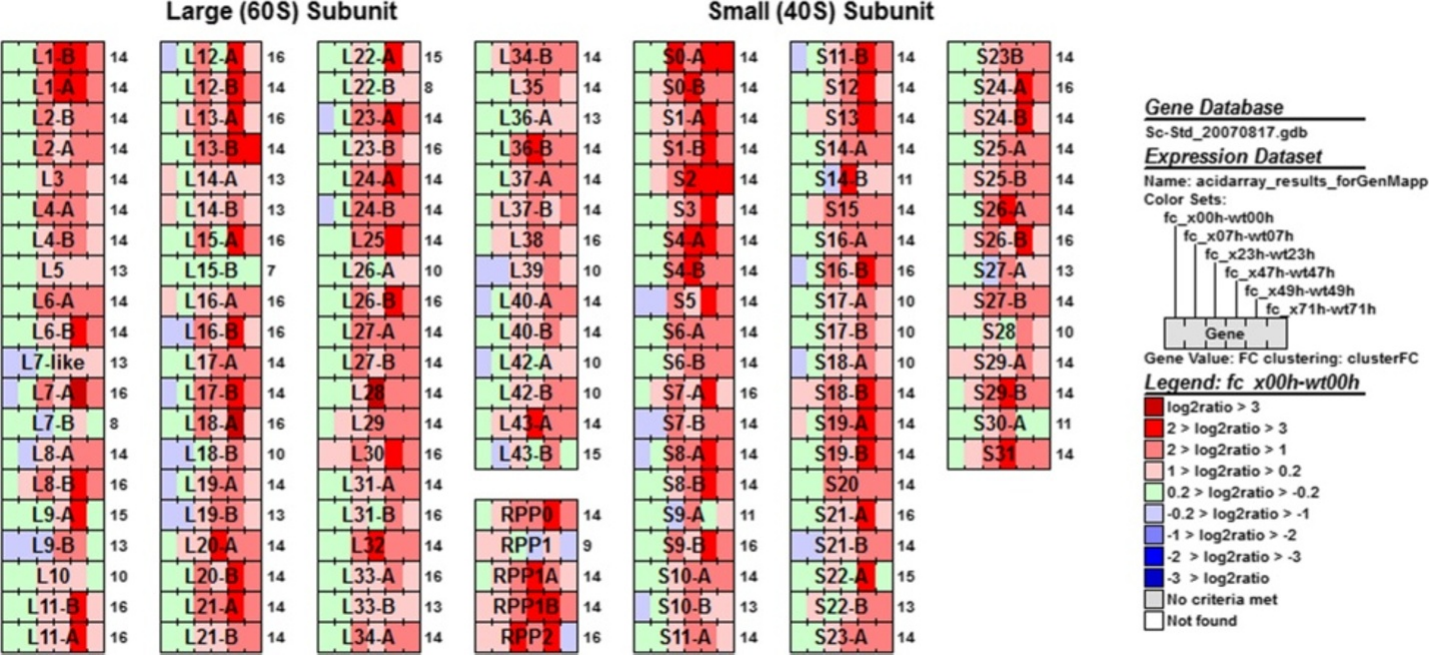
**Genes encoding ribosomal proteins.** Visualization of log2 ratios of fold changes in gene expression between the xylonate producing and control strain during time. Comparisons were made for samples taken from the D-xylonate producing strain at 0, 7, 23, 47, 49 and 71 h, compared to the corresponding time point in the control strain, with time progressing left to right in each bar, as indicated in the legend. Darker red denotes a larger difference. The legend for the colours is as in Figure 4. The number next to each protein denotes the FC cluster the gene belongs to. The visualization was created using the GenMAPP tool [21].

Clusters NP11, NP13, and NP25 contain genes whose expression were either increased or not downregulated with time in the D-xylonate producing strain, *i.e.* as D-xylonate accumulated in the culture (Figure 2A). FC11, FC13, and FC14 clusters contain the genes with relatively higher expression in the D-xylonate producing strain than in the control (Figure 2B). In these six clusters, there were a significant proportion of genes encoding proteins involved in processes related to metabolism of proteins such as translation, RNA metabolism, ribosomal structure, and protein degradation. Enrichment analysis of the clusters verified this conclusion: gene ontology (GO) categories related to protein biosynthesis were significantly enriched in these clusters. For example, GO:0006412 (translation) was enriched with p-value = 2.2e-55 in cluster FC14 and GO:0044257 (protein catabolic process) was enriched with p-value 6.1e-27 in cluster FC11. Visualisation of the log2 ratio profiles (used as the basis of FC clustering) of all genes encoding ribosomal proteins illustrates the higher expression of translation machinery during D-xylonate production compared to the absence of production (Figure 3).

Clusters NP11, NP13, NP25, FC11, FC13 and FC14, with genes that either had increased expression or were not down-regulated, contained *HAA1* (NP25, FC11), *TPO2* (NP13, FC14), *PMA1* (NP11, FC14), *PDR12* (NP13, FC8), *PDR5* (NP11, FC13), *SSA4* (NP25, FC11) and genes encoding components of vacuolar membrane proton pumps, such as *VMA10*, *TFP3*, *VPH1*, and *VPS3. HAA1* and *TPO2*, encoding a transcription factor and a drug:H^+^ antiporter, respectively, play an important role in acetic and propionic acid tolerance [13, 14]. *PMA1* encodes a plasma membrane proton pump from the ABC-family that plays an essential role in pH homeostasis [22, 23]. *PDR12* and *PDR5* also encode multidrug transporters and are involved in responses to stresses induced by various organic acids [10, 24–28]. *SSA4* is induced by sorbate stress [10]. *HSP30* encodes a plasma membrane protein induced by multiple stresses, including weak organic acids [10, 29]. The vacuolar membrane proton pumps are also crucial for intracellular pH regulation. These genes have previously been reported to be significantly up-regulated during weak acid stress (reviewed in [17]).

**Figure 4:**
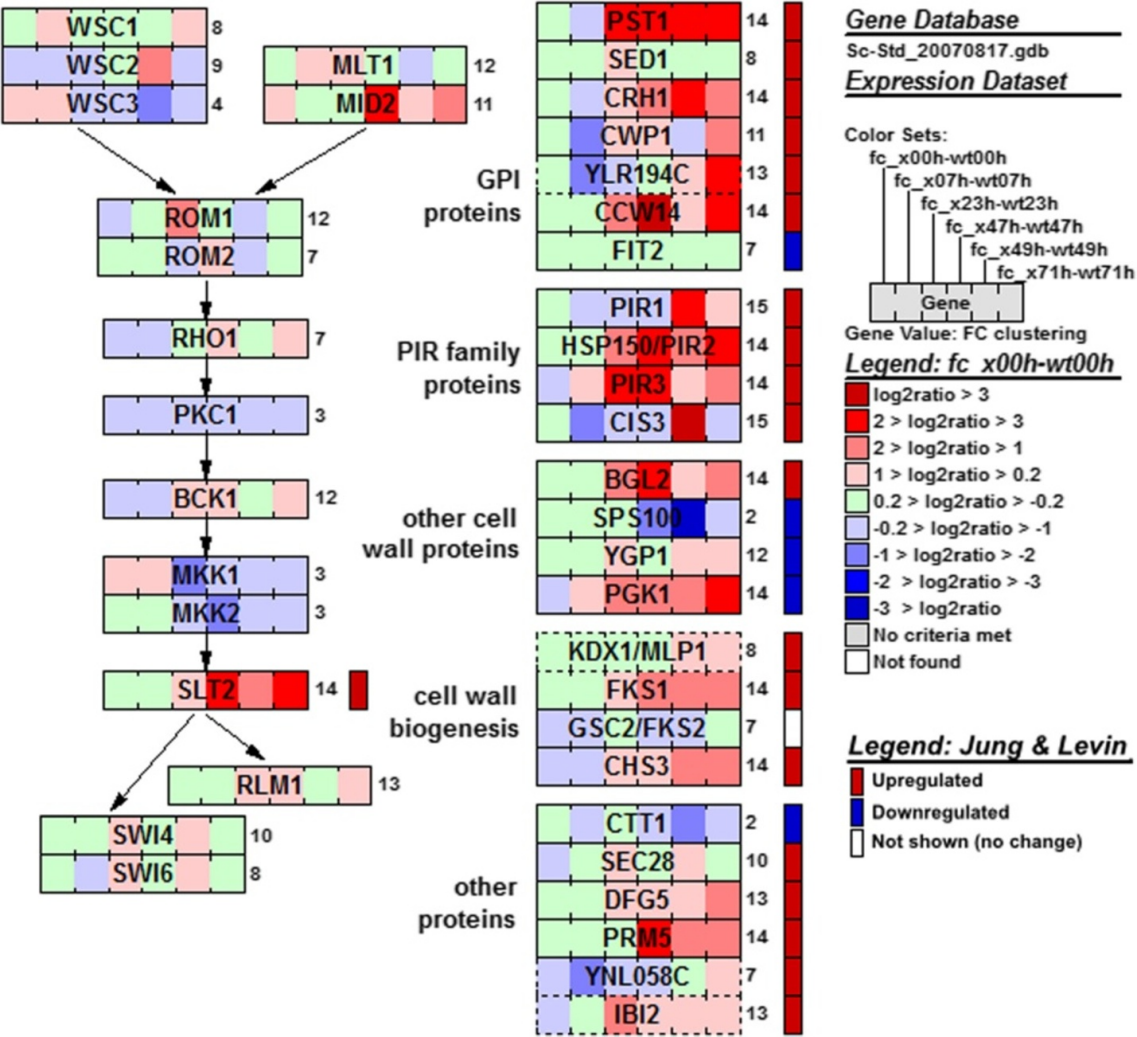
**Genes positively regulated by the cell wall integrity pathway.** Visualization of log2 ratio between the D-xylonate producing and the control strains. The number next to each gene denotes the FC cluster the gene belongs to. The visualization was created using the GenMAPP tool [21]. The cell wall integrity pathway was drawn based on [30, 31]. 21 genes previously reported [30, 31] to be positively regulated by the CWI pathway are indicated with red boxes to the right of each gene; genes negatively regulated are indicated with blue.

A significant portion of genes involved in cell wall biosynthesis, the cell wall integrity (CWI) pathway, and protein glycosylation were also present in these clusters. Almost all genes regulated by the PKC-SLT2 MAP-kinase (cell wall integrity) pathway, as identified by Jung and Levin [30], were members of these clusters. Sixteen out of 20 genes positively regulated by the CWI pathway were significantly more expressed, and two out of five genes negatively regulated by the CWI pathway were significantly less expressed during D-xylonate production than in the control strain, especially in the later stages of cultivation (Figure 4).

### Delayed response in D-xylonate producing strain at 23 h

Clusters NP3 and NP9 Figure 2C) showed differences in the responses of the two strains at 23 h and 49 h. Many genes encoding glycolytic enzymes (*PGI1, FBA1, TPI1, TDH2, ENO 1/2, CDC19, PDC1, ADH1, ADH3*) were present in cluster NP9 (GO:0006096, glycolysis, was enriched with p-value 0.00076), and many TCA cycle and related genes (*PDA1, CIT1/2, ACO1, KGD2, SDH1/3, ICL1, MLS1, GOR1, IDP2, SFC1*, etc.) were in cluster NP3 (GO:0006099, TCA cycle, was enriched with p-value 3.6e-08). The transcription profile of cluster NP9 indicated that the glycolytic genes were already down-regulated in the control strain at 23 hours (but not yet at 7 hours), which corresponded to the absence of D-glucose at this time. These genes were still relatively highly expressed in the D-xylonate-producing strain at 23 h, even though D-glucose had been consumed at a similar rate as by the control strain (Figure 1). A similar trend was seen at 49 h, when the glycolytic genes were down-regulated in the control strain, in comparison to the D-xylonate-producing strain (Figure 2C). The transcription profiles in cluster NP3 showed a similar shift in the timing of expression responses of other metabolic genes. In NP3, the level of transcription of genes encoding enzymes of the TCA cycle and processes related to post-diauxic shift (respiratory growth) were significantly overrepresented. While in the control strain, the genes of TCA cycle had already been down-regulated at 23 hours and up-regulated at 49 hours, in the D-xylonate-producing strain these genes were actively transcribed at 23 hours, but not yet up-regulated at 49 hours (Figure 2C).

### Groups of genes with lower expression during D-xylonate production than in the control

Genes with lower relative expression levels in the D-xylonate producing strain than in the control were pooled in clusters FC1 and FC3 (Figure 2D). Cluster FC1 only contained 72 genes and interestingly included the genes *PDR10*, encoding a multidrug ABC transporter regulated by Pdr1 [32], and *PMA2*, encoding an isoform of the plasma membrane H^+^-ATPase Pma1p. A large percent (25%) of genes in cluster FC3 encoded proteins involved in the transport of various molecules, both through intracellular membranes and through the plasma membrane. In addition, cluster FC3 contained genes involved in autophagy and negative regulation of the PKA pathway (*IRA1*, *IRA2* and *RGS2*); GO:0006914, autophagy, was enriched with p-value 0.0014. Comparison of this cluster with the corresponding NP clusters (NP15 and NP20) revealed that many of the genes were in fact upregulated in both strains between 23 and 71 hours, but that their expression was either delayed or significantly lower in the D-xylonate-producing strain (Figure 2E).

### Validation of micro-array data

#### Expression analysis of selected genes during batch growth in flasks

The results of the transcriptomics analysis were validated by analysing the expression of some genes which had shown dominant differences in the microarray analysis in batch flask cultures that corresponded to the first phase of the bioreactor cultures (i.e. the first 47 hours, before addition of D-glucose and D-xylose), but lacked pH control. The accumulation of biomass was almost identical in the D-xylonate producing strain and the control strain, but consumption of D-glucose was slightly slower in the D-xylonate producing strain. Ethanol utilisation was slower in flasks than in the bioreactor, reflecting poorer aeration in the flasks, but the production strain again consumed ethanol more slowly than the control. After an initial pH decrease in both cultures, the pH increased in the control cultures, but continued to decrease in cultures of the production strain, as D-xylonate accumulated (Additional file 5).

**Figure 5:**
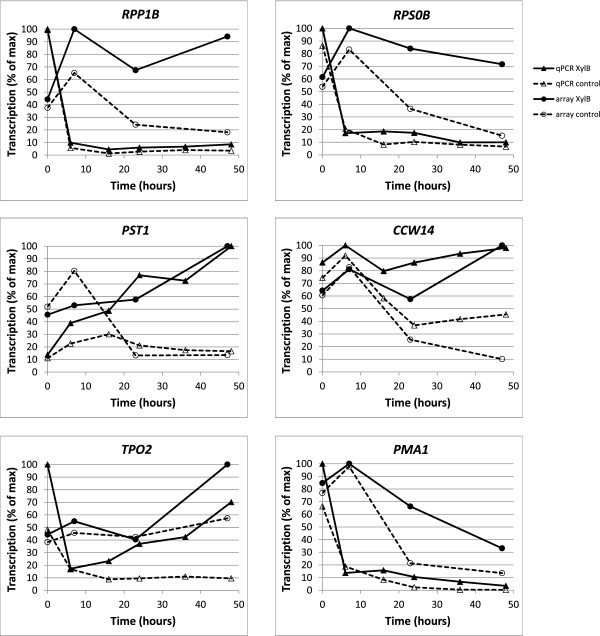
**Comparison of qPCR and microarray analyses of selected genes.** Expression profiles of translation-related genes (*RPP1B* and *RPS0B*, top panel), genes controlled by the cell wall integrity pathway (*PST1* and *CCW14*; middle panel), and acid stress-associated genes (*TPO2* and *PMA1*, lower panel). The comparison of the two methods shows strong similarity of the profiles from corresponding conditions. The apparently different patterns for the *RPP1B*, *RPS0B*, and *PMA1* genes are caused by a strong upregulation of these genes in presence of D-glucose, which is captured in the qPCR “0 h” time-point, but not in the microarray “0 h” time-point. Recalculated profiles, considering only the conditions without D-glucose, show significant similarity for the profiles of these genes also (Additional file 8). The shown values represent the average of two (qPCR) and three (microarray) independent cultivations.

The expression profiles of genes analysed by PCR from flask cultures were similar to those observed with array analysis from the bioreactors (Figure 5). Higher expression of translation-related genes during D-xylonate production was shown with *RPP1B* and *RPS0B*. The cell wall integrity pathway activation was represented by *PST1* and *CCW14*. And elevated acid stress associated responses were represented by *TPO2* and *PMA1*.

### Dynamics of the CWI pathway in response to D-xylonate production

The response of the cell wall integrity pathway to the later phase of D-xylonate production was assessed by analysing the phosphorylation of the MAP kinase Slt2, which was increasingly more strongly phosphorylated in the *xylB* expressing strain than in the control after 16 hours of D-xylonate production (Additional file 6). This correlated with the transcription profiles of genes which are under control of the CWI pathway. Slt2 is activated upon phosphorylation and in turn phosphorylates transcription factor Rlm1 which is the key activator of the CWI pathway genes [33] (Figure 4). In both the control and the D-xylonate producing strain, Slt2 was transiently phosphorylated during the diauxic shift.

### Possible role of D-xylonate accumulation in deregulation of the PKA pathway

Intracellular concentrations of cAMP did not differ between the control and the production strain (not shown). However, the levels of trehalose and glycogen in the strains differed considerably (Additional file 6). The lowest level of trehalose in both strains was observed during early logarithmic growth, at 2 hours. After this, the level increased and then decreased in both strains, but was markedly lower in the D-xylonate producing than in the control strain from 6 to 36 h. Glycogen levels were comparable in the two strains during the first 6 hours of the cultivation, after which more glycogen was observed in the D-xylonate producing strain than in the control between 16 and 24 hours, but less after 36 h (Additional file 6).

### Comparison to published acid production and acid stress transcriptomic data

**Figure 6:**
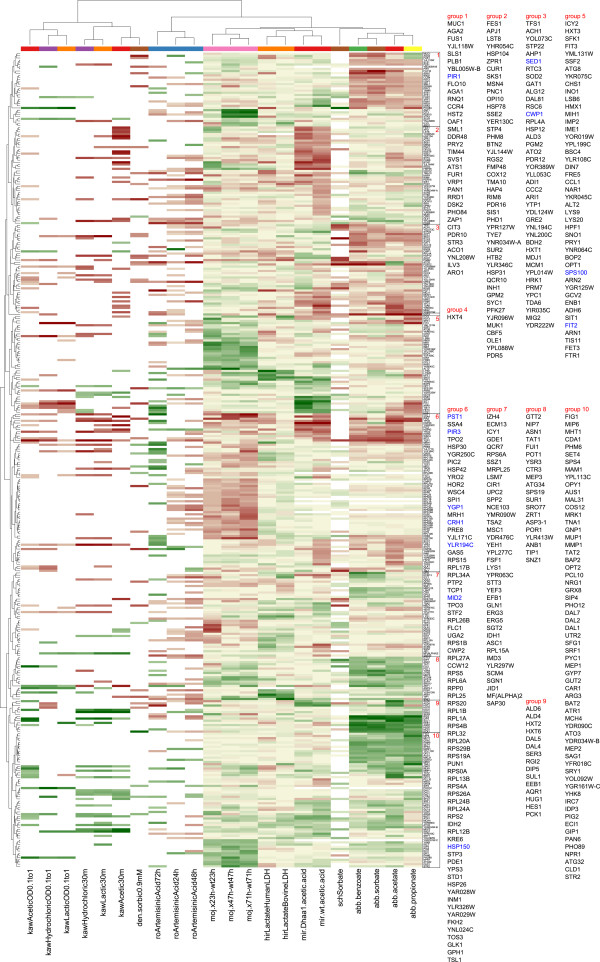
**Comparison of transcriptional responses in different weak acid stress and acid production conditions.** Previously published microarray data on weak acid tolerance and acid production (Table 1) were combined, normalized together, and clustered. This heat map includes genes that were reported to be significant in at least two of the comparisons. Cell wall integrity pathway genes (Figure 4) are indicated in blue in the lists of genes. Gene names are provided in a bigger font, group by group, in the same order as they appear in the rows of the heat map. The coloured bar above the columns of the heat map (just below the column dendrogram) indicates samples which involved the same acid. Column descriptions can be found in Table 1, where they are listed in the order that they appear in this clustering (left to right).

Transcriptomic data obtained in this study were compared to transcriptomic data from published acid production and acid stress experiments in *S. cerevisiae* (Table 1). The combined data-set had 22 comparisons, including three from this study (Table 1). A subset of 375 genes that were reported to be significant in at least two of the comparisons was selected. The resulting gene expression data matrix was clustered using hierarchical clustering and visualized as a heatmap (Figure 6). No general weak acid response was observed in all experiments, i.e. different weak acids induce different transcriptional responses. However, common responses at the level of individual genes were observed for some of the acids. In addition, the experimental setup was found to have a strong impact on the responses; experiments with the same acid in different publications did not group together. In cases with similar experimental setups, the responses were mostly similar (*e.g.* 30 min samples of acetic acid stress [14, 34]). On the other hand, genes with common biological functions responded similarly to different acids: the GO categories enriched (p < 0.01) in the selected list of acid-responding genes were transmembrane transport (51 genes), lipid metabolism (40 genes), translation (30 genes), amino acid metabolism (25 genes), cell wall organization (21 genes), iron ion homeostasis (12 genes) and cell adhesion (5 genes).

**Table 1:** **List of publications and brief description of the conditions they included, used in the comparison of transcriptional responses shown in Figure** 6

	Condition	Control	pH	Publication
kaw acetic OD 0.1 to 1 (adaptation)	0.3% w/v acetate (OD = 0.1), growth until OD = 1	WT strain, no acid	pH 3.2	Kawahata et al. [34]
kaw hydrochloric OD 0.1 to 1 (adaptation)	0.03% w/v hydrochloric acid (OD = 0.1), growth until OD = 1	WT strain, no acid	pH 2.5	Kawahata et al. [34]
kaw lactic OD 0.1 to 1 (adaptation)	0.3% w/v lactate (OD = 0.1), growth until OD = 1	WT strain, no acid	pH 2.8	Kawahata et al. [34]
kaw hydrochloric 30 m (shock)	30 min after addition of 0.03% w/v hydrochloric acid	WT strain, no acid	pH 2.6	Kawahata et al. [34]
kaw lactate 30 m (shock)	30 min after addition of 0.3% w/v lactate	WT strain, no acid	pH 2.8	Kawahata et al. [34]
kaw acetic 30 m (shock)	30 min after addition of 0.3% w/v acetate	WT strain, no acid	pH 3.3	Kawahata et al. [34]
den sorbic 0.9 mM	late exponential stage, 0.9 mM sorbate	WT strain, no acid	pH 4.5	de Nobel et al. [35]
ro artemisinic 72 h	artemisinic acid-producing strain 72 h	strain with inactivated gene 72 h	pH 5.5	Ro et al. [24]
ro Artemisinic 24 h	artemisinic acid-producing strain 24 h	strain with inactivated gene 24 h	pH 5.5	Ro et al. [24]
ro Artemisinic 48 h	artemisinic acid-producing strain 48 h	strain with inactivated gene 48 h	pH 5.5	Ro et al. [24]
moj x23h-wt23h	Xylonate production, after 23 h	WT strain, no production, after 23 h	pH 5.5	This publication
moj x47h-wt47h	Xylonate production, after 47 h	WT strain, no production, after 47 h	pH 5.5	This publication
moj x71h-wt71h	Xylonate production, after 71 h	WT strain, no production, after 47 h	pH 5.5	This publication
hir lactate human LDH	Lactate production using Human LDH 16 h	WT strain, no lactate production	pH 5	Hirasawa et al. [36]
hir lactate bovine LDH	Lactate production using Bovine LDH 16 h	WT strain, no lactate production	pH 5	Hirasawa et al. [36]
mir DHaa1 acetate acid	haa1 deletion strain, 30 min in 50 mM Acetate	haa1 deletion strain, no acid	pH 4	Mira et al. [14]
mir wt acetic acid	WT strain, 30 min in 50 mM Acetate	WT strain, no acid	pH 4	Mira et al. [14]
sch sorbate	WT strain, 20 min in 8 mM Potassium Sorbate	WT strain, no acid		Schuller et al. [10]
abb benzoate	WT strain, steady state at 0.27 mM Benzoate	WT strain, no acid	pH 5	Abbott et al. [18]
abb sorbate	WT strain, steady state at 0.47 mM Sorbate	WT strain, no acid	pH 5	Abbott et al. [18]
abb acetate	WT strain, steady state at 37.7 mM Acetate	WT strain, no acid	pH 5	Abbott et al. [18]
abb propionate	WT strain, steady state at 8.6 mM Propionate	WT strain, no acid	pH 5	Abbott et al. [18]

The overall transcriptional response of *S. cerevisiae* during D-xylonate production was different from that in any other acid production or acid stress experiment (Figure 6). Some similarity was, however, observed between responses to D-xylonate and artemisinic acid production, in which the pattern of translation-related genes expression was shared.

### Comparison to published general stress data

The transcriptome data obtained from bioreactor cultures in this study were also compared to data from yeast general stress response studies [37, 38]. The combined data-set had 168 comparisons, including ten from this study (five comparisons for the D-xylonate producing strain, and five comparisons for the control strain). A subset of 1006 genes was selected: the environmental stress response (ESR) genes identified in the Gasch et al. study [37], genes that responded at least three fold in at least 5 of the experiments in Causton et al. [38] and finally, genes that were significantly differentially expressed in the D-xylonate producing strain at 23 h compared to 0 h or at 47 h compared to 0 h. The resulting gene expression data matrix was clustered using hierarchical clustering and visualized as a heatmap (Additional file 7).

A typical environmental stress response (ESR) was seen in most stress experiments (nutrient depletion, hyper osmotic or heat shock, oxidative or pH stress, etc.), with the interesting exceptions of hypo-osmotic shock and mild cold stress, which had opposite responses to other stresses (Additional file 7). D-Xylonate production did not cause a typical ESR, but three comparisons from this study, 7 h vs. 0 h in both strains and 49 h vs. 0 h in the D-xylonate producing strain, showed similarities in the overall gene expression pattern with hypo-osmotic shock (90 and 120 minutes after shock from Causton et al. [39], and 60, 90 and 120 minutes after shock from Gasch et al. [40]) and mild cold stress. Some of the other comparisons from this study showed some similarities with conditions tested by Gasch [40] and Causton [39] that had less distinct ESR responses. Most of the comparisons from this study from later times in the cultivations (47 h, and 71 h in both strains and 23 h and 49 h in the control strain) vs. 0 h clustered together and were generally similar in terms of their global expression pattern. The only exception was the 23 h vs. 0 h comparison for the D-xylonate producing strain, in which the Rap1 target genes [41] were not down-regulated or were even up-regulated. In general, the later times vs. 0 h comparisons showed down-regulation of most of the genes that are typically downregulated in general (environmental) stress, except for the Rap1 target genes, which were not down-regulated in any D-xylonate comparisons. In contrast, only a fraction of the typically upregulated ESR genes were also upregulated during the cultivations performed in this study.

## Discussion

Production of more than 20 g/L D-xylonate is a challenge to *S. cerevisiae* in terms of intracellular acid accumulation and viability [9], which could limit the development of industrially relevant processes with high D-xylonate production. In order to understand how cells respond to producing D-xylonate, we analysed the transcriptome of this strain during D-xylonate production. Transcriptional profiling revealed that only some of the genes previously identified as involved in a weak acid stress response were differentially expressed in the D-xylonate producing strain, in comparison to the control strain. In addition, genes involved in the cell wall integrity pathway, translational machinery and protein catabolism were significantly more expressed. However, some of the genes encoding proteins involved in transmembrane transport and autophagy were down-regulated. D-Xylonate production did not cause a typical environmental stress response (ESR), either. Although production of D-xylonate led to unexpected expression of many genes which are part of the ESR, the expression profile differed from previously described responses to various stresses.

The integrated analyses of transcriptomic data which had originated from different publications and/or from different microarray platforms provided clear evidence that acid induced stress differs from the general ESR and is much more diverse. Comparison of such diverse data required normalisation, to ensure that all data was compared on the same basis, and selection of an appropriate subset of genes (feature selection). Although only a subset of genes was used for the integrated microarray data visualizations (Figure 6, Additional file 7), the selection of genes did not change the main conclusion that producing D-xylonate is not very similar to other stress conditions, including stress from other weak acids or production of other acids.

The generally unique transcriptional response during D-xylonate production, relative to transcriptional responses to other weak acids or acid production, was in accordance with previous studies which have shown that the chemical structure of the weak acid strongly affects transcriptional responses [17, 18]. Our integrative data analysis also showed that responses towards even the same acids may be different when the culture conditions and strain backgrounds are different. Even though different individual genes respond to different weak acids, they do share the same biological functions: protein folding, lipid metabolism, cell wall function, and multidrug resistance [17]. In addition to these, our analysis showed that there are also changes in transmembrane transport, amino acid metabolism, iron ion homeostasis, and cell adhesion. During D-xylonate production, we observed changes in the transcription of individual genes in all these categories, but only in the case of cell wall function and transmembrane transport was a large group of genes involved.

The cell wall integrity (CWI) pathway was activated during the later stages of D-xylonate production, as seen both in the transcription of nearly all CWI-regulated genes (Figure 4), and in the phosphorylation of Slt2 kinase. Normally, the CWI pathway is activated in conditions which cause stress or directly damage the cell wall [31, 39, 40, 42, 43] *.* The CWI pathway is also regulated during morphological changes, in a cell cycle dependent manner, and by mating pheromone [44, 45]. Slt2 phosphorylation has previously been shown to be activated by low pH caused by addition of HCl and by acetic acid stress [46, 47]. The activation of this pathway during D-xylonate production thus indicates that cells producing D-xylonate experience stress on their cell walls, either directly from the D-xylonate or indirectly as a consequence of other cellular stress caused by its production. The nature of damage to the cell wall caused by low pH or weak acid stress is not known. Since cell wall related genes have also been observed to be up-regulated under other weak acid stress conditions, it may be inferred that reinforcement of the cell wall is a general response to weak acids [17] (Figure 6).

Translation-related genes are usually repressed under environmental stresses such as nutrient depletion, heat shock and osmotic shock [37]. For example, translation-related genes are already repressed at the time of D-glucose depletion, before the diauxic shift [48]. However, the D-xylonate producing strain had higher expression levels of these genes than the control strain, even though the cells were apparently under such severe stress that cell viability was reduced. Down-regulation of the major translation-related genes did occur in both production and control cultures as a result of D-glucose depletion, but to a lesser extent in the D-xylonate producing strain, so that subsequently it had higher relative expression of these genes (Figure 5, Additional File 8). This, together with an increase in the abundance of genes encoding proteins involved in proteolysis, indicated that there was increased protein turn-over in the D-xylonate-producing strain compared to the control, especially in the later stages of the cultivation. A similar response in transcription of translation-related genes has been observed in cells producing artemisinic acid [24]. The viability of the cells during artemisinic acid production was not reported, but plasmid stability was shown to be very low, which may indicate that the artemisinic acid production also caused stress to the cells. However, apart from the translation-related genes, the transcriptional responses caused by the production of D-xylonic acid and artemisinic acid were not similar.

Upon nutrient limitation, yeast cells normally enter stationary phase, during which they are more resistant to environmental stress. The process of entry to the stationary phase is not fully understood, but the transcriptional changes involved happen already before and during the diauxic shift [49]. Then synthesis of trehalose and glycogen are activated via a mechanism involving the protein kinases Rim15 and PKA [50]. While glycogen is thought to be primarily a storage carbohydrate, the level of trehalose is associated with stress resistance. The level of both of these carbohydrates was considerably different in the D-xylonate-producing strain than in the control. We also observed significantly lower expression of genes involved in autophagy, the process cells use for recycling of proteins and cellular organelles and which is required for survival during nutrient limitation (and stationary phase), in the D-xylonate producing strains than in the control. There was a pattern of increased budding in the later stage of D-xylonate production (data not shown). These observations (level of trehalose and glycogen, expression of autophagy related genes, and budding pattern) in combination with the loss in viability during D-xylonate production indicated that cells producing D-xylonate were unable to adapt normally to stationary phase. In this respect D-xylonate producing cells resemble cells with constitutive PKA pathway activation, which do not adapt to stationary phase upon nutrient deprivation, show low level of storage carbohydrates, are highly sensitive to heat shock and grow poorly on non-fermentable and weakly fermentable carbon sources [51, 52].

The PKA pathway is one of the major regulators of the cell cycle and of general stress responses [53], but D-xylonate producing cells appear to have an activated PKA pathway during stationary phase, when they should not. Acidification of the cytosol, which has been observed in D-xylonate producing cells [9, 54], may lead to accumulation of cAMP and activation of the PKA pathway [51, 53, 55], although measurable increase in cAMP levels was not observed in D-xylonate producing cells. None-the-less, some of the negative regulators of the PKA pathway (*IRA1*, *IRA2* and *RGS2*) were less expressed in the production than in the control strain, and the lack of down-regulation of Rap1 targets [41] (Additional file 7) may also reflect PKA activation. Thus, the transcriptional analysis, along with the physiological data, showed that activation of the PKA pathway may play an important role during later stages of D-xylonate production by *S. cerevisiae*, while the inability to adapt to stationary phase may contribute to the observed loss of viability of the production strain.

## Conclusions

Weak organic acids have pleiotropic effects on the physiology of *S. cerevisiae*. In contrast to the common transcriptional response to most environmental stresses, this and previous studies of transcriptomic responses in *S. cerevisiae* to the presence or production of various organic acids have shown that there is no general transcriptional response to all organic acids, but rather both the structure of the acid and the experimental conditions determine the response. The transcriptional response to D-xylonate production was unique, differing from both general stress responses and from responses induced by other organic acids.

Production of D-xylonic acid leads to intracellular accumulation of the acid and dramatically decreased viability [9]. D-Xylonate production apparently imposed considerable stress on the cell wall. However, there was also evidence that increased protein turnover, imbalance in pH homeostasis, and activation of the PKA pathway occurred during D-xylonate production, leaving cells unable to adapt normally to stationary phase. This, together with intracellular acidification, probably contributed to cell death.

## Methods

### Strain and culture conditions

An industrial strain of *S. cerevisiae*, B67002 (VTT Culture Collection) and B67002 *xylB*, containing two copies of *xylB* under the *PGK1* promoter [9] were used in this study. Cells were grown in bioreactors (three independent cultures for each strain) in YP medium containing 10 g/L yeast extract and 20 g/L bacto-peptone at pH 5.5, 30°C, 500 rpm and 0.5 vvm aeration, as previously described [9]. The initial amounts of D-glucose and D-xylose were 8 g/L and 21 g/L, respectively. Cultures were inoculated with approx. 0.4 g/L biomass (OD_600_ 1.5). After D-xylose had been consumed by the *xylB*-expressing strain, at 47 hours, D-glucose (4 g/L) and D-xylose (~25 g/L) were added to the cultures. Metabolites, biomass and viability were measured as described previously [9].

To validate observations from the bioreactor cultures, the strains were grown in 1 L Erlenmeyer flasks in 400 mL of YP medium containing 10 g/L D-glucose and 20 g/L D-xylose at 30°C, 250 rpm. The initial optical density at 600 nm (OD_600_) was ~1.

### Microarray analysis

Cells were harvested in 2 volumes of cold sodium phosphate buffer (0.1 M, pH 7), collected by centrifugation (2 min. at 3600 rpm, 4°C), frozen in liquid nitrogen and stored at -80°C. Total RNA was isolated using the RNeasy Plant Mini Kit (QIAGEN), according to the glass-bead method. The microarray analysis was performed as described in the Nimblegen microarray manual (Nimblegen). The quality of the RNA and cDNA preparations was assessed using a Bioanalyser 2100 (Agilent Technologies). Labelled cDNA was hybridized on *S. cerevisiae* 12x135K chip arrays (Nimblegen). The hybridization, washing and other procedures were performed according to the Nimblegen Arrays User’s guide. An MS 200 Microarray scanner was used to scan the chips, and the fluorescent intensities of the probes were extracted using the accompanying software (MS 200 Data Collection Software; Nimblegen).

R/Bioconductor, version 2.10.1 (R Development Core Team, 2008, http://www.r-project.org) was used for all data analysis. The raw data was normalized with Robust Multichip Average (RMA) normalization [56]. The quality of the microarray data was assessed based on a report from the arrayQualityMetrics –package [57] and by looking at the distribution of the log2 intensities on each array before and after normalization. Additionally, the similarity of replicate samples (3) to each other was verified by plotting the arrays on a two dimensional display using principal component analysis.

Statistical differences in expression were analysed using linear modelling with tools of the limma package [58]. For each gene, a linear model was fitted by the least squares method and differential expression within pairs of experimental conditions was computed using an empirical Bayesian approach [59]. The Benjamini & Hochberg method for controlling false discovery rate (FDR) [60] was used for correction of multiple testing errors.

The clustering analysis of gene expression data was performed using fuzzy c-means clustering [20], implemented in the R-package Mfuzz. This clustering method assigns genes to clusters with gradual membership (values between 0 and 1). For clustering based on a normalized profile (NP), the expression values were scaled and centred to have a mean of zero and standard deviation (or variance) of 1.

For clustering based on fold-change (FC), the log2 ratio of gene expression between the two conditions was computed. This gives equal weight to up- and down- regulation, with minus one indicating 2 fold down-regulation and plus one 2 fold up-regulation in the D-xylonate producing strain compared to the control strain. The two parameters of the algorithm were set after repeated runs of the algorithm: m controls the sensitivity of the clustering process to noise and c the number of clusters. For selecting m, the algorithm was run on a randomized data set with various values of m and c. Parameter m was set to 1.35 to prevent the detection of clusters in the randomized data. For selecting c, the algorithm was run on the real data with m = 1.35 and various values of c. The number of clusters was selected such that no clusters were formed in which all the genes would have membership values below 0.5, and such that similar looking clusters (by visual inspection) were formed in repeated runs of the algorithm. The genes were not filtered before the fuzzy c-means clustering. The enriched GO classes and KEGG metabolic pathways in the clusters were computed with the GOstats package [61]. Visualizations of gene expression fold changes on metabolic pathways and gene sets were done using the GenMAPP tool [21]. Heatmap visualizations of microarray data were created in R using the heatmap.plus package (with slight modifications to make more compact dendrograms) and the marray package to create the green to red colour scheme. Additional labels were added to the figures using the Inkscape vector graphics editor.

### Integrative analysis of microarray data from multiple publications

#### Combination of acid production and weak acid stress studies

In order to compare our microarray data to previously published studies of transcriptomic responses to weak acid stress and acid production (Table 1) and to general stress response data [37, 38], the data for each published study was downloaded and then processed in R/Bioconductor. The data was transformed to log2 ratios (log2 of the fold-change) between acidic and non-acidic conditions, or in the case of general stress responses to log2 ratios between late and time zero samples, and then normalized to the same range across all studies. The use of log2 ratios comparing samples of interest with appropriate control samples removes some of the experiment-specific sources of variation between experiments, but results in different ranges of fold change for different conditions. To normalize the arrays, Quantile normalization was applied to datasets with full data [14, 18, 36], and the log2 ratios from publications listed in Table 1 were scaled to the same range [-4.03, 4.07]. As a result, the exact fold-changes reported in the original source were lost, but the transformation provided similar log2 ratios for the most significantly changed genes in each study, keeping the rank or order of genes with transcriptional responses in a given condition the same. Without quantile normalization some experiments would have dominated the analysis (data not shown), but some information was lost: i.e. it was no longer possible to verify whether a larger range of log2 ratios resulted from a larger fold change in mRNA levels, or from differences in experimental design between experiments.

The list of significant genes was extracted from the original publication when possible, or significance tests were conducted using the same/similar thresholds as in the original publication. The analysis steps for the data sources included (Table 1; [37, 38]) are described in the supplementary material (Additional file 9).

The log2 ratios from previously published studies on acid tolerance and production were combined with our microarray data from *S. cerevisiae* producing or not producing D-xylonate (at 23 h, 47 h or 71 h after start of production).

The combined data was clustered using hierarchical clustering (Euclidean distances, complete linkage algorithm). Only genes that were considered significant in at least two comparisons (*i.e.* in at least two of the various lists of significant genes mentioned above), and measured in our microarrays, were included in the clustering result (375 genes). Note that an additional 446 genes were significant in at least one of the D-xylonate production comparisons (p-value cut-off 1e-05, log2ratio cut-off 1, Benjamini & Hochberg method controlling false discovery rate [60]), but were not included in this analysis.

### Combination of xylonate production and general stress studies

Data from this study (for the control and D-xylonate producing strains at 7, 23, 47, 49 and 71 h) and from Causton et al. [38] were transformed to a log2 ratio between a later time and time zero of the corresponding experiment. For example, log2 ratios were determined for data at 47 h relative to 0 h in the D-xylonate production cultivation (this study) or for data at 45 minutes after the heat shock, relative to immediately before it [38]. Data in Gasch et al. [37] had been published as normalized log2 ratios. The combined data was then normalized to same range across all comparisons using quantile normalization.

The combined data was clustered using hierarchical clustering (Euclidean distances, complete linkage algorithm). Only genes that were considered significant at 23 h or 47 h after inoculation, compared to 0 h, or genes that were identified as environmental stress response (ESR) genes in Gasch et al. [37] were included. The list of common environmental response (CER) genes from Causton et al. [38] was no longer available at the supplementary information site, so only genes responding in at least 5 of the time series in the Causton data were included. A cut-off of three fold induction/reduction was used, as in the original publication. Any genes that were not included in our microarrays were discarded and we then used a set which contained 1006 genes.

For comparisons with both the acid stress and the ESR stresses, other selections schemes were also tested, including a larger or smaller fraction of the differentially expressed genes. The visualizations were selected that best conveyed the conclusions which were observed in other visualisations which included various numbers of genes.

### Quantitative PCR

To quantify the transcription of selected genes, cells from two independent cultures were collected at the times indicated in the Results, washed with ice-cold water and stored at -80°C. Total RNA was purified according to the RNeasy® Plant Mini Kit protocol 1c for yeast (QIAGEN). Samples were normalised based on the total RNA concentration prior to the cDNA synthesis, since housekeeping genes such as *ACT1* and *IPP1* were observed in the microarrays to change considerably between phases of growth on D-glucose or ethanol, stationary phase and accumulation of D-xylonate and could therefore not be used as internal standards. 4 μg of purified total RNA was used for production of cDNA in the reverse transcription reaction (Transcriptor First Strand cDNA Synthesis Kit; Roche). The cDNA was diluted 1:20 with water and 2.5 μL of diluted solution was used in qPCR reactions (LightCycler 480 SYBR Green I Master; Roche, Switzerland). The primers are listed in Table 2. The reactions were carried out in a LightCycler 480 Instrument II (Roche, Switzerland) and the analysis was performed with the accompanying software. The relative abundance of the template was calculated from the C_t_ values and shown as percentage of the maximal expression during the experiment.

**Table 2:** **Primers used for qPCR analysis**

RPP1A_qPCR_F	AATCCGCTTTGTCTTACGCC
RPP1A_qPCR_R	CGCTGAAGTTGACCAATAAGTC
RPL28_qPCR_F	ACAAGCAACAAGCTCATTTCTG
RPL28_qPCR_R	GACGATAACTGGAACATTTGGA
PST1_qPCR_F	CCACATCTGTTAAACTATCGTCC
PST1_qPCR_R	ATAGACATGATGATTGCCGT
CCW14_qPCR_F	TCCTTCCAGTGAAGAATCCT
CCW14_qPCR_R	CTAGAACATTACCAGAACCTTCAG
TPO3_qPCR_F	CTGAAGATCGTTTGCTAGGT
TPO3_qPCR_R	TCAACACCATACCGAAACCA
PMA1_qPCR_F	GTCCATTCTGGTCTTCTATCCC
PMA1_qPCR_R	GCTTACCGTTCATCAATCTGTC

### Slt2 phosphorylation

Identical amount of cells were collected from two independent cultures for each strain at different times, as indicated in the Results. The cells were collected by centrifugation, washed with ice-cold water, and stored at -80°C. The cell pellets were resuspended in 0.3 mL of 2 M NaOH with 7% β-mercaptoethanol and incubated on ice for 5 minutes for complete cell lysis. Proteins were precipitated by addition of 0.3 mL of 50% TCA. The proteins were sedimented by centrifugation and washed (after resuspension) in 1 M Tris–HCl (pH = 8.0). The supernatant was discarded and the resulting protein pellets were solubilized in SDS-loading buffer at 80°C for 20 minutes. Equal amounts of proteins were loaded onto an SDS-polyacrylamide gel (Criterion TGX 4-20%; BioRad), in duplicate. The proteins were blotted onto a nitrocellulose membrane (0.2 μm; BioRad) and the equivalent loading was confirmed by staining with Ponceau S (Sigma-Aldrich). Total Slt2 was detected by Mpk1 (yC-20): sc-6803 antibody (Santa Cruz Biotechnology) and Anti-Goat-AP-conjugate antibody (Sigma-Aldrich). Phosphorylated Slt2 was detected by Phospho-p44/42 MAPK (Erk1/2) (Thr202/Tyr204) Antibody (Cell Signaling Technology) and Goat-anti-Rabbit AP-conjugate antibody (BioRad). The signals were quantified on a GS-710 Calibrated Imaging Densitometer (BioRad). Signals from phosphorylated Slt2 were divided by the corresponding signals from total Slt2 protein to obtain relative amounts.

### Trehalose and glycogen

10 mg of cells (dry weight) were collected from four independent cultures by centrifugation (3000 rpm, 3 minutes) and washed with water. The cells were treated with 250 μL Na_2_CO_3_ for 4 hours at 95°C. The solution was brought to pH 5.2 by adding 150 μL 1 M acetic acid, and then 600 μL of 0.2 M Na-acetate. 500 μL of this solution was incubated overnight with 0.05 U/mL of trehalase (Sigma, T8778) at 37°C to release D-glucose. Another 500 μL of the solution was incubated with 1–2 U/mL of amyloglucosidase (Sigma, A7420) at 50°C to release D-glucose. The D-glucose released was measured using a D-glucose oxidase kit (Sigma, GAGO20).

### Availability of supporting data

The raw and processed microarray data can be accessed through GEO accession GSE52736.

## Electronic supplementary material

Additional file 1: **NP-clusters, showing 25 clusters of individual gene expression profiles based on normalized values from the D-xylonate producing and the control strain during time.** (PDF 283 KB)

Additional file 2: **FC-clusters, showing 16 clusters of individual gene expression profiles based on fold-changes between the D-xylonate producing and the control strain during time.** (PDF 118 KB)

Additional file 3: **Comparison of the two clustering approaches.** The table shows the number of genes that a NP cluster and a FC cluster had in common. The clusters visualized in Figure 2 are indicated with blue and orange shading. The biggest number of genes shared with a FC cluster is indicated in bold for each NP cluster and vice versa. (PDF 46 KB)

Additional file 4: **Combined data for all genes and their membership to the clusters.** The dataset includes input data used for clustering and the descriptions of individual gene/protein functions. (XLS 6 MB)

Additional file 5: **Physiological data from flask cultivations.** A) Growth (OD_600_) and pH. The measurements were performed in quadruplicates; average ± standard deviation is shown. B) Extracellular concentrations of D-glucose, D-xylose and ethanol as determined by HPLC. The experiments were performed in duplicates and the average values are shown. (PDF 86 KB)

Additional file 8: **Modified comparison of qPCR and microarray analyses of** ***RPP1B*** **,** ***RPS0B*** **, and** ***PMA1*** **genes.** Data from 0 h has been removed from the expression profiles to remove the impact of D-glucose induction which was observed in qPCR data, but not in bioreactors. The values represent the average of two (qPCR) or three (microarrays) independent cultivations. (PDF 67 KB)

Additional file 6: **Physiological responses during the D-xylonate production.** A) Phosphorylation of the Slt2 kinase. The left panel shows the average of two (or 4, for 16–48 h) densitometry analyses with ± standard deviation; the right panel shows a section of representative western blot used for densitometry. B) Trehalose content in cells, as determined by analysis of D-glucose released by trehalase treatment. C) Glycogen content in cells, as determined by analysis of D-glucose released by amyloglucosidase treatment. Data in B and C were obtained from 4 independent flasks. (PDF 128 KB)

Additional file 7: **Overall visualization of stress-related gene expression patterns (1006 selected genes) from this study, from Gasch et al.** [37] **and from Causton et al.** [38]. (PDF 1 MB)

Additional file 9: **Description of the pre-processing of the microarray data from other publications.** (DOCX 24 KB)
