# Supplementary figures and images for: Transcriptome of Saccharomyces cerevisiae during production of D-xylonate
Source: BMC Genomics. 2014 Sep 5;15(1):763. doi: 10.1186/1471-2164-15-763 (PMC4176587; doi:10.1186/1471-2164-15-763)

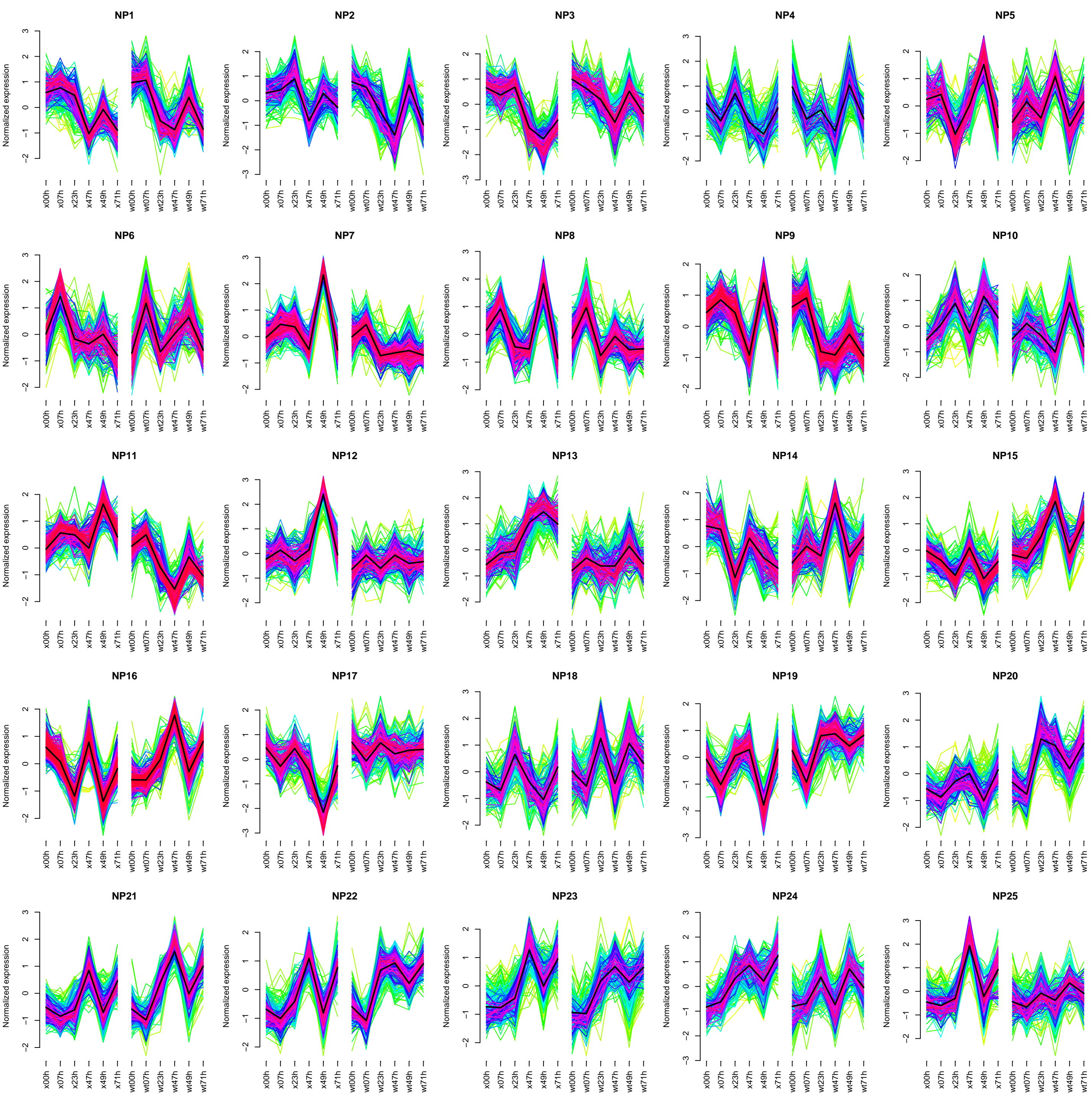

Supplement: Supplementary file 1 — Additional file 1: NP-clusters, showing 25 clusters of individual gene expression profiles based on normalized values from the D-xylonate producing and the control strain during time. (PDF 283 KB) [file 12864_2014_6465_MOESM1_ESM.pdf]

**FC1**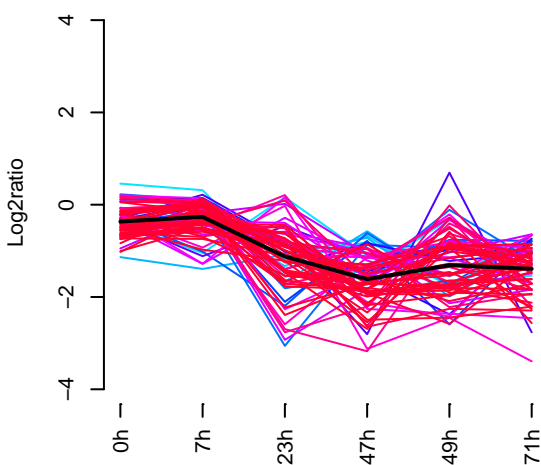**FC2**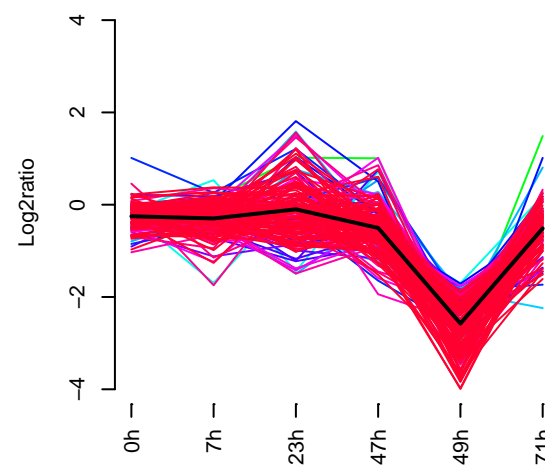**FC3**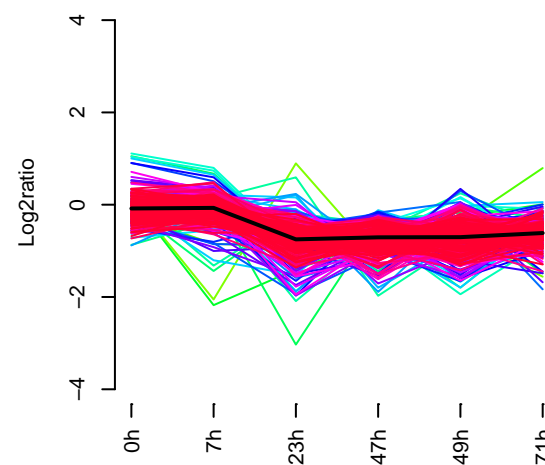**FC4**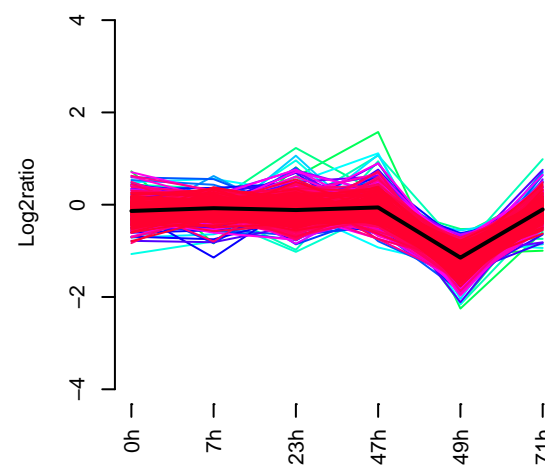**FC5**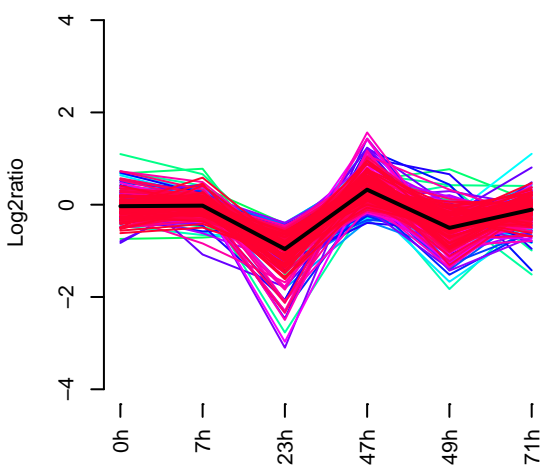**FC6**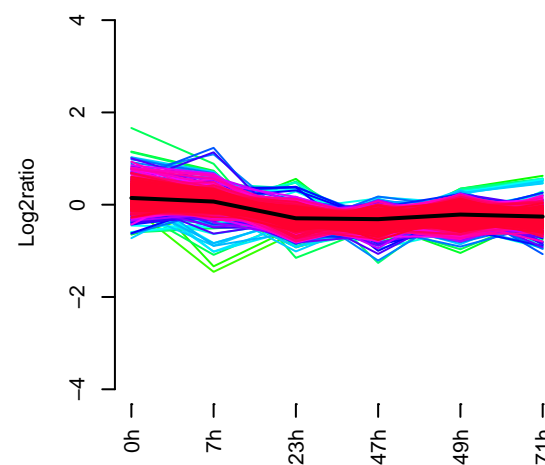**FC7**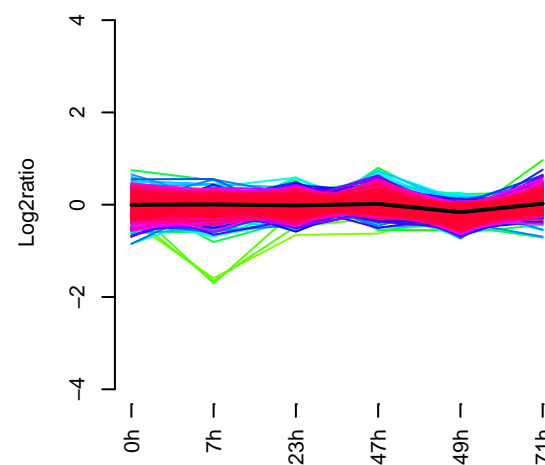**FC8**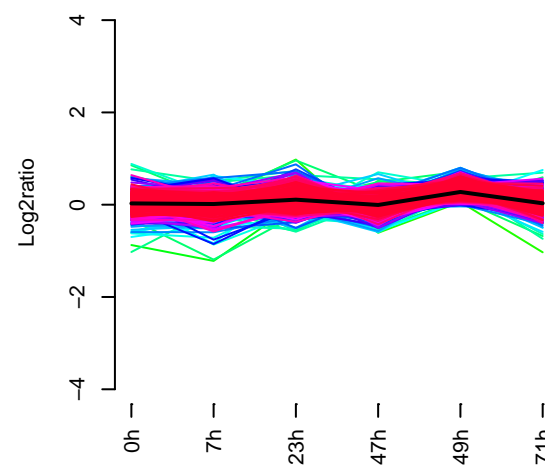**FC9**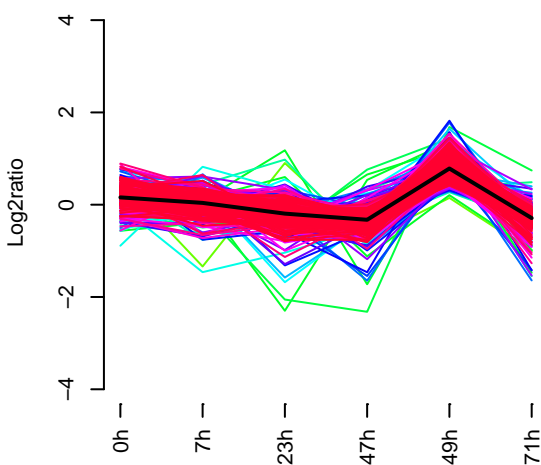**FC10**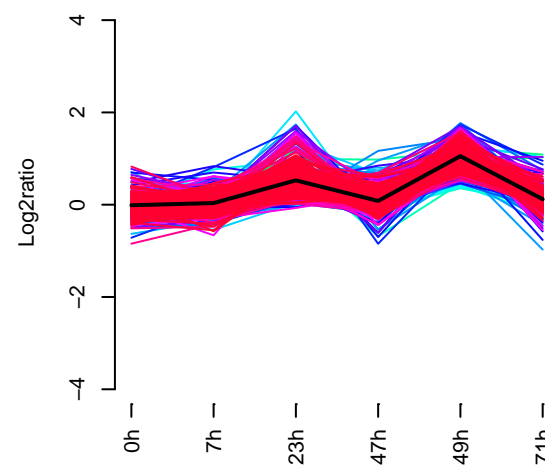**FC11**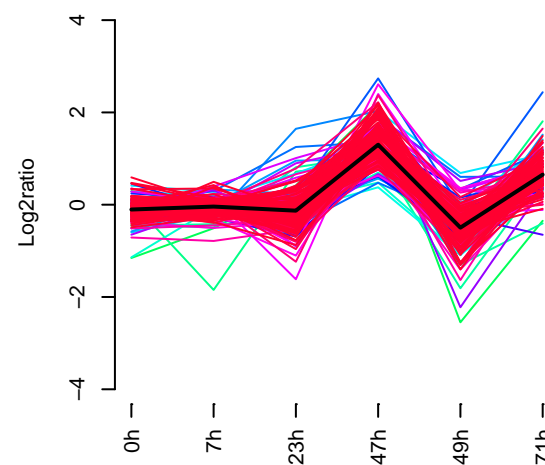**FC12**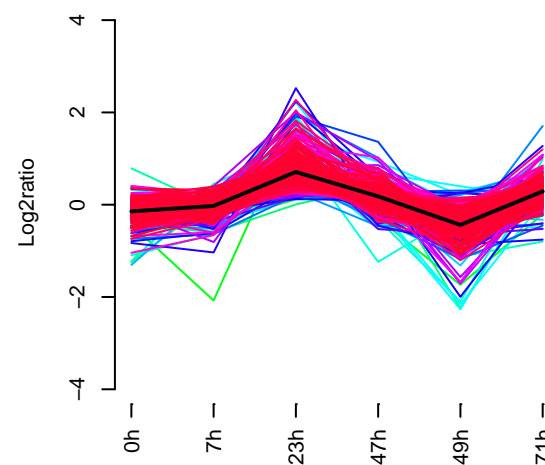**FC13**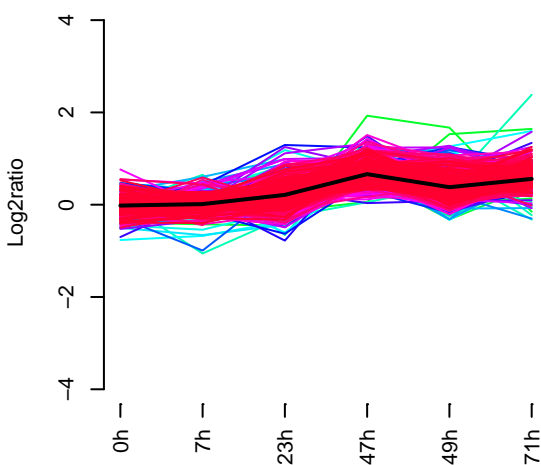**FC14**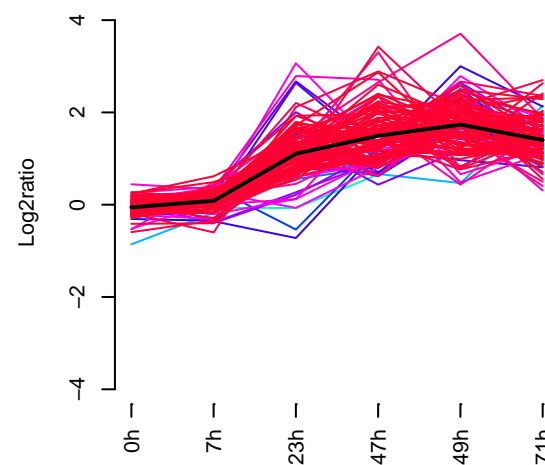**FC15**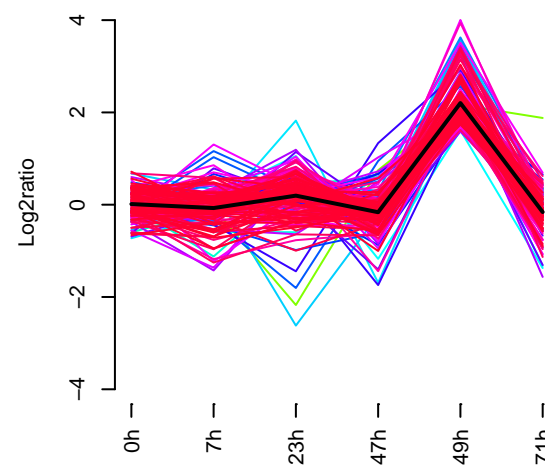**FC16**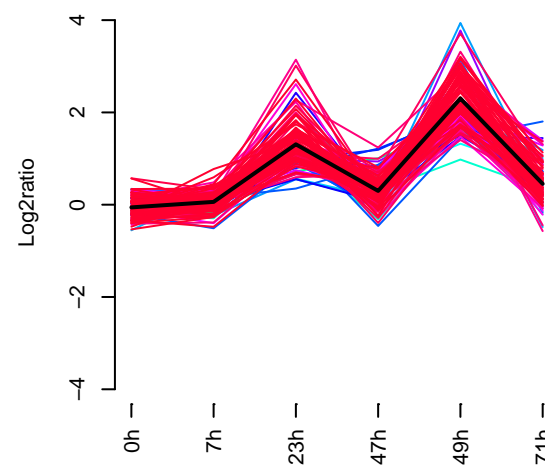

Supplement: Supplementary file 2 — Additional file 2: FC-clusters, showing 16 clusters of individual gene expression profiles based on fold-changes between the D-xylonate producing and the control strain during time. (PDF 118 KB) [file 12864_2014_6465_MOESM2_ESM.pdf]

A.

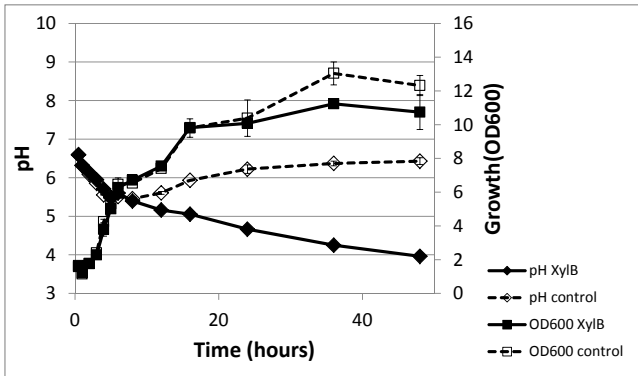

B.

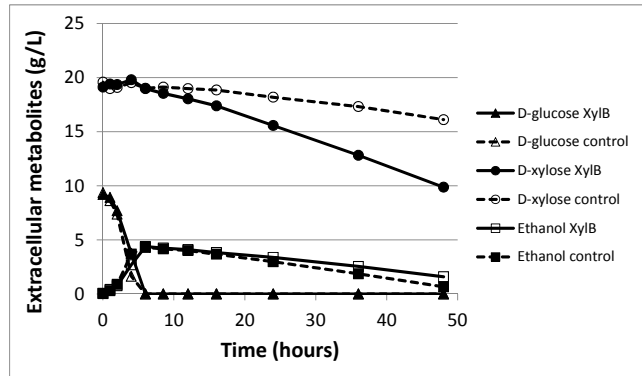

Supplement: Supplementary file 5 — Additional file 5: Physiological data from flask cultivations. A) Growth (OD600) and pH. The measurements were performed in quadruplicates; average ± standard deviation is shown. B) Extracellular concentrations of D-glucose, D-xylose and ethanol as determined by HPLC. The experiments were performed in duplicates and the average values are shown. (PDF 86 KB) [file 12864_2014_6465_MOESM5_ESM.pdf]

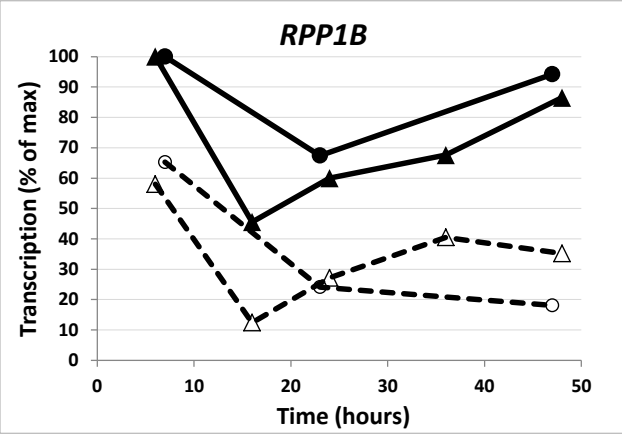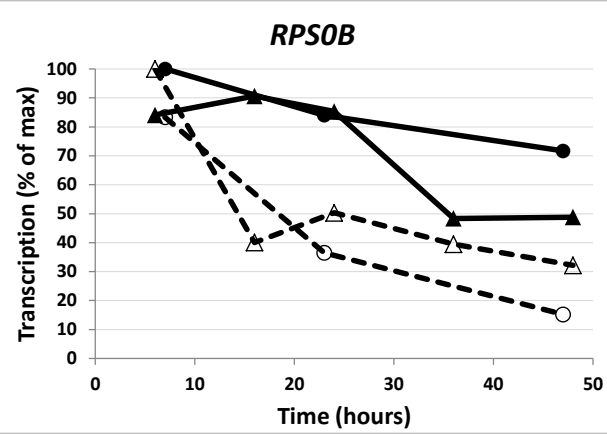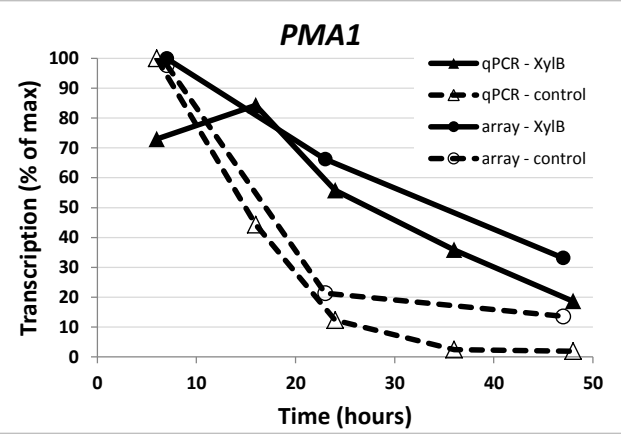

Supplement: Supplementary file 6 — Additional file 8: Modified comparison of qPCR and microarray analyses of RPP1B , RPS0B , and PMA1 genes. Data from 0 h has been removed from the expression profiles to remove the impact of D-glucose induction which was observed in qPCR data, but not in bioreactors. The values represent the average of two (qPCR) or three (microarrays) independent cultivations. (PDF 67 KB) [file 12864_2014_6465_MOESM6_ESM.pdf]

**A - Slr2 phosphorylation**

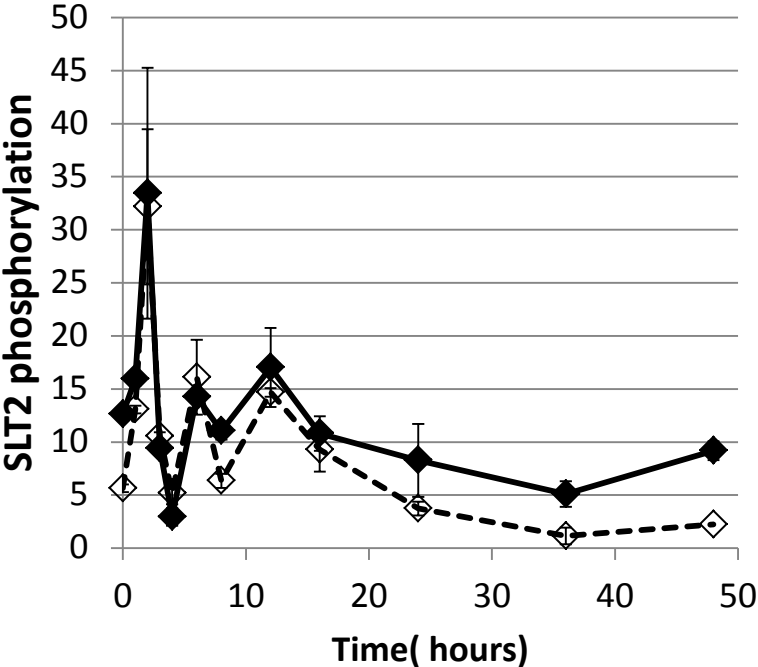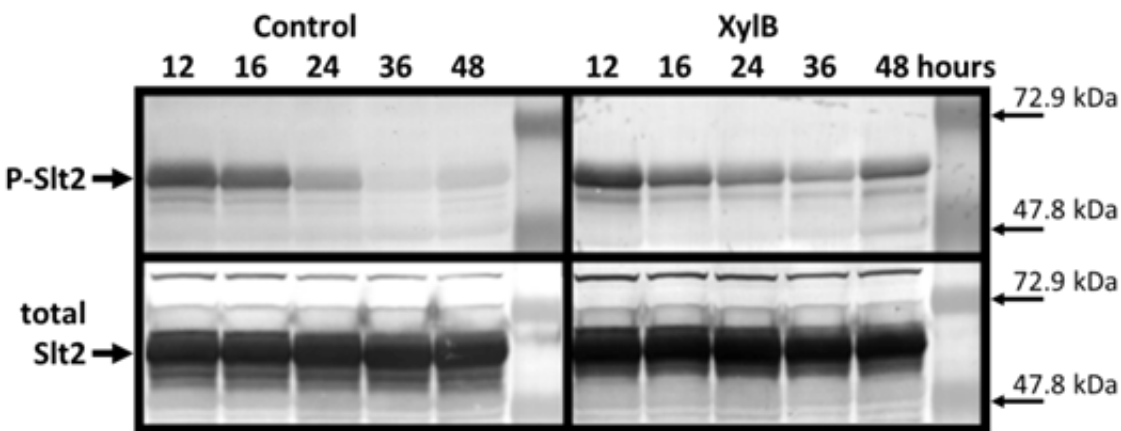

**B - Trehalose content**

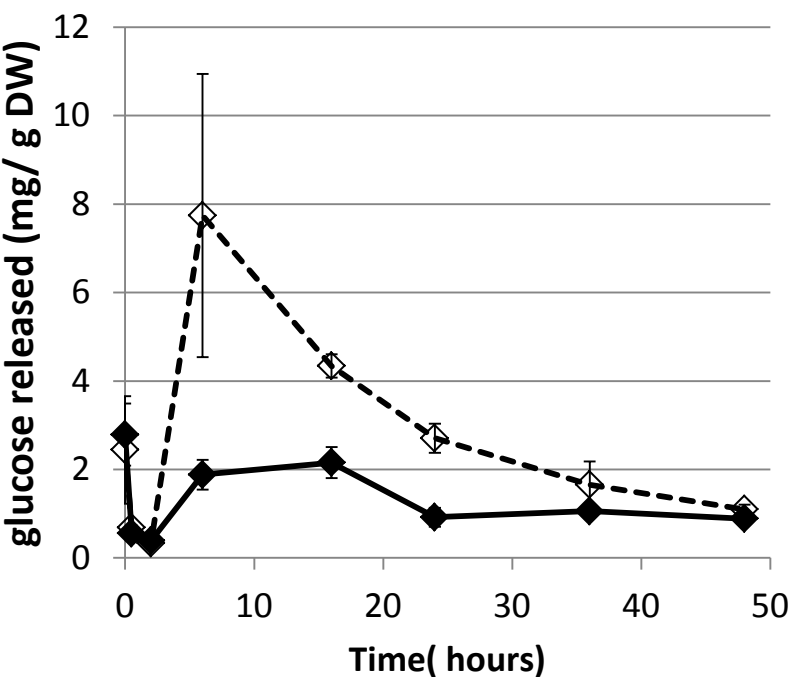

**C - Glycogen content**

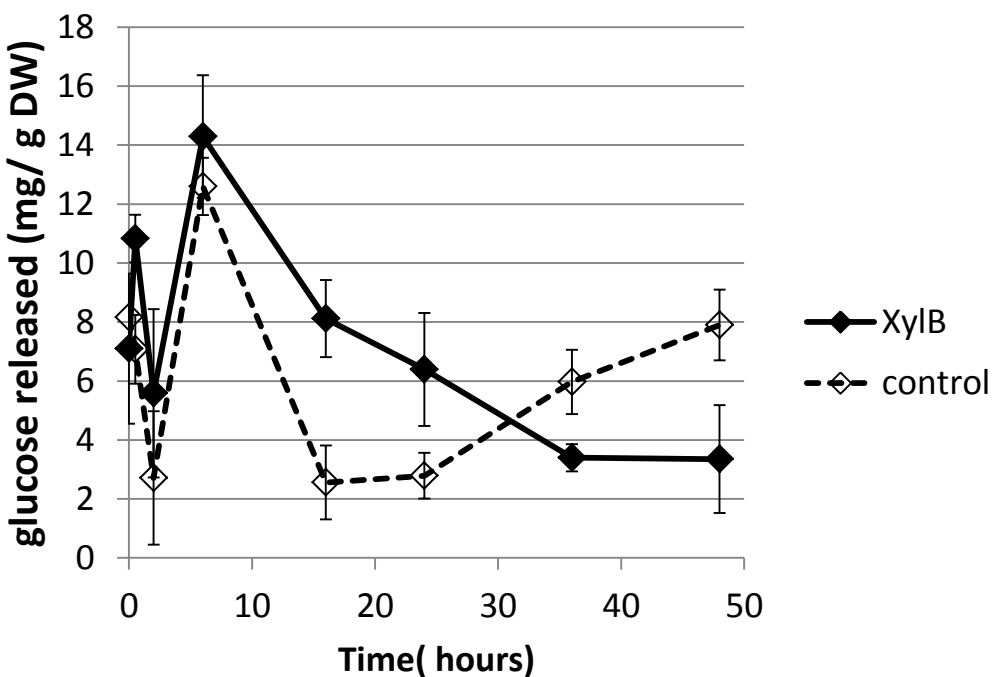

Supplement: Supplementary file 7 — Additional file 6: Physiological responses during the D-xylonate production. A) Phosphorylation of the Slt2 kinase. The left panel shows the average of two (or 4, for 16–48 h) densitometry analyses with ± standard deviation; the right panel shows a section of representative western blot used for densitometry. B) Trehalose content in cells, as determined by analysis of D-glucose released by trehalase treatment. C) Glycogen content in cells, as determined by analysis of D-glucose released by amyloglucosidase treatment. Data in B and C were obtained from 4 independent flasks. (PDF 128 KB) [file 12864_2014_6465_MOESM7_ESM.pdf]
