# Supplementary material for: Transcriptome of Saccharomyces cerevisiae during production of D-xylonate
Source: BMC Genomics. 2014 Sep 5;15(1):763. doi: 10.1186/1471-2164-15-763 (PMC4176587; doi:10.1186/1471-2164-15-763)
Supplement: Supplementary file 3 — Additional file 3: Comparison of the two clustering approaches. The table shows the number of genes that a NP cluster and a FC cluster had in common. The clusters visualized in Figure 2 are indicated with blue and orange shading. The biggest number of genes shared with a FC cluster is indicated in bold for each NP cluster and vice versa. (PDF 46 KB) [file 12864_2014_6465_MOESM3_ESM.pdf]

Number of genes common between a FC and a NP cluster

FC clusters

NP clusters	1	2	3	4	5	6	7	8	9	10	11	12	13	14	15	16
**1**	0	0	1	10	1	10	**86**	34	4	0	1	**74**	2	0	0	0
**2**	0	0	0	0	1	0	55	32	0	2	13	**81**	35	3	0	0
**3**	1	30	8	**69**	1	20	37	4	2	0	4	26	0	0	0	0
**4**	0	1	1	25	0	3	**86**	2	0	0	13	43	7	0	0	0
**5**	0	0	0	0	0	8	14	**71**	**87**	5	0	0	0	0	2	0
**6**	0	0	2	7	1	32	**111**	28	4	1	0	5	3	0	0	0
**7**	0	0	0	0	0	0	1	**98**	13	**112**	0	0	0	0	32	36
**8**	0	0	0	0	0	1	5	**90**	**71**	28	0	0	2	2	**55**	5
**9**	0	0	0	0	0	0	4	**106**	4	**114**	0	5	8	5	26	**57**
**10**	0	0	0	0	0	0	**60**	**70**	2	0	3	26	23	0	0	0
**11**	0	0	0	0	0	0	0	25	0	21	3	0	**86**	**97**	2	17
**12**	0	0	0	0	0	0	7	**102**	27	44	0	0	5	2	26	1
**13**	0	0	0	0	0	0	20	53	0	8	4	3	**96**	11	1	0
**14**	0	0	0	0	1	**114**	45	29	14	0	0	0	0	0	0	0
**15**	10	0	**58**	0	1	**115**	39	1	3	0	0	0	0	0	0	0
**16**	0	0	15	4	10	**199**	130	4	1	0	0	1	0	0	0	0
**17**	13	**56**	16	**66**	2	18	39	0	0	0	1	0	0	0	0	0
**18**	1	2	10	46	20	22	**84**	1	1	0	1	6	0	0	0	0
**19**	14	**53**	26	**87**	7	15	46	0	0	0	0	1	0	0	0	0
**20**	**27**	1	**73**	2	23	57	24	4	11	0	0	0	0	0	0	0
**21**	9	1	52	4	11	**91**	58	11	12	0	0	0	0	0	0	0
**22**	1	4	16	32	**106**	17	76	0	0	0	4	1	0	0	0	0
**23**	0	0	0	4	26	12	**110**	43	17	9	7	3	12	0	1	0
**24**	0	0	0	1	3	0	**100**	24	0	1	35	16	37	0	1	0
**25**	0	0	0	2	6	0	**83**	6	0	0	**83**	6	19	0	0	0
