# Supplementary material for: Transcriptome of Saccharomyces cerevisiae during production of D-xylonate
Source: BMC Genomics. 2014 Sep 5;15(1):763. doi: 10.1186/1471-2164-15-763 (PMC4176587; doi:10.1186/1471-2164-15-763)

Comparison of transcriptional responses in stress experiments and in xylonate production.

This heatmap visualization contains a subset of 1006 genes responding to stress in Gasch et al, in Causton et al, or showing differential expression in this study between xylonate production and control strain (23h or 47h). The left most column indicates previously identified environmental stress respose (ESR) genes. Genes that are typically down-regulated during stress are indicated in green, and typically up-regulated genes are indicated in red. The next column indicates RAP1 target genes. Ribosomal RAP1 target genes are shown in red, non-ribosomal targets are shown in orange, and the other ribosomal genes (not targets of RAP1) are indicated in cyan. The main block of RAP1 target genes, that show unexpected behaviour in our experiments are indicated by boxes on top of the heatmap visualization. ESR genes typically upregulated in stress, but not upregulated in the later time point comparisons from this study are also indicated by a box on top of the heatmap visualization.

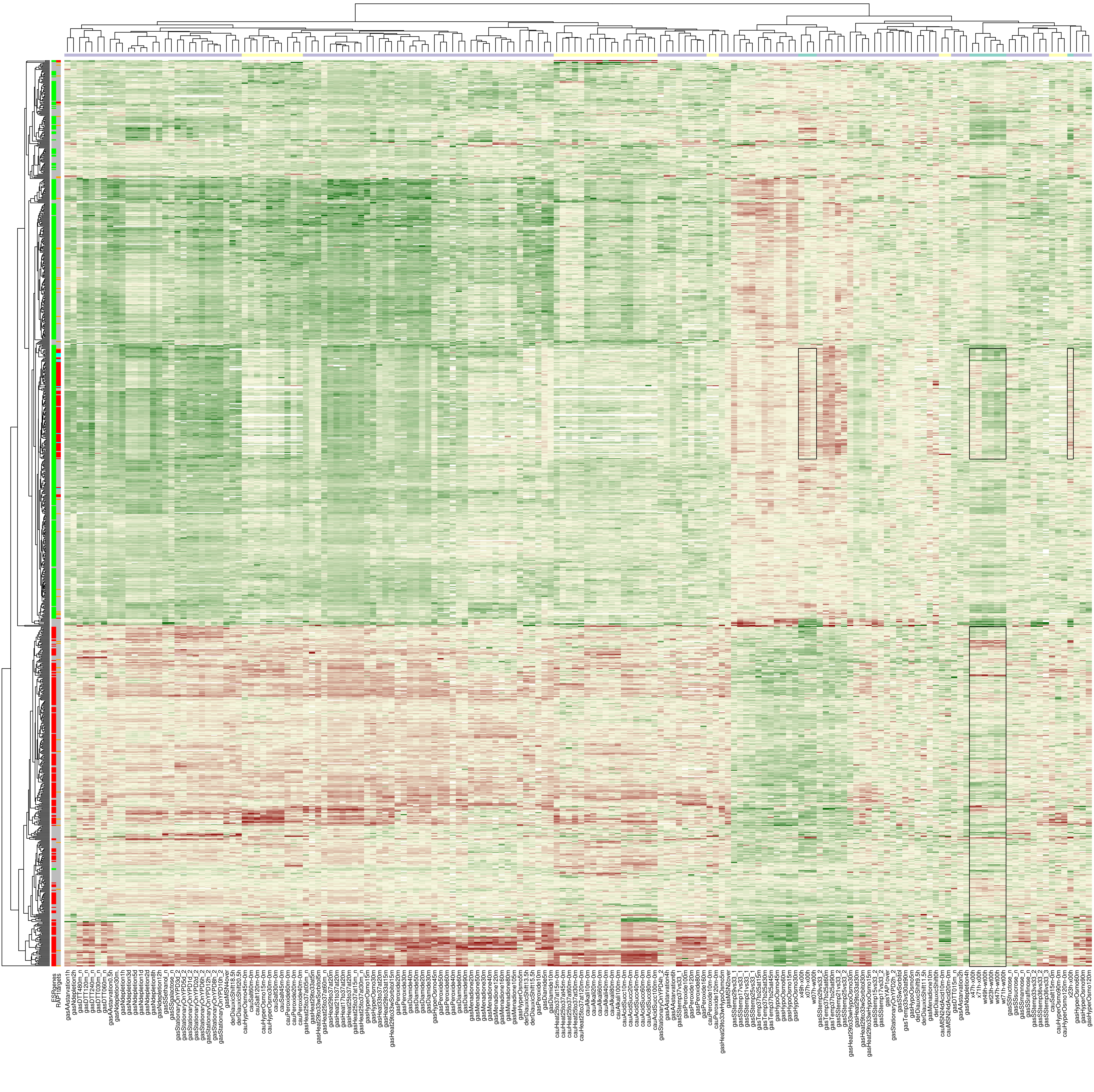

Supplement: Supplementary file 8 — Additional file 7: Overall visualization of stress-related gene expression patterns (1006 selected genes) from this study, from Gasch et al. [37] and from Causton et al. [38]. (PDF 1 MB) [file 12864_2014_6465_MOESM8_ESM.pdf]
