# Supplementary material for: Transcriptome of Saccharomyces cerevisiae during production of D-xylonate
Source: BMC Genomics. 2014 Sep 5;15(1):763. doi: 10.1186/1471-2164-15-763 (PMC4176587; doi:10.1186/1471-2164-15-763)
Supplement: Supplementary file 9 — Additional file 9: Description of the pre-processing of the microarray data from other publications. (DOCX 24 KB) [file 12864_2014_6465_MOESM9_ESM.docx]

Processing public microarray data

# Details on processing the microarray data from this publication

**Significant genes:** For combining our data to previously published gene expression data sets, significance tests were conducted using the “limma” package in R/Bioconductor. P-value threshold was 0.00001, and the FDR [Benjamini and Hochberg, 1995] method was used for adjusting the p-values for the purpose of correcting for multiple testing. The significance tests were conducted without fold change threshold.

# General stress response data sets

## Gasch et al

Gasch AP, Spellman PT, Kao CM, Carmel-Harel O, Eisen MB, Storz G, Botstein D, Brown PO: **Genomic expression programs in the response of yeast cells to environmental changes**. *Mol Biol Cell* 2000, **11**(12):4241-4257.

**Retrieving data:** The complete data set was downloaded from <http://genome-www.stanford.edu/yeast_stress/index.shtml>. The data was provided in a single text file containing "normalized, background-corrected **log2** values of the Red/Green **ratios** measured on the DNA microarrays".

**Post normalization**: for the HeatShock25to37, DTT, and carbon source series the data was "zero normalized" after loading the supplementary data file: As described in the article the data of the later time points (other carbon sources) were divided by the data for the zero time point (glucose). In addition, the first time series of these experiments were discarded, because the time points (carbon sources) didn't match to those listed in the article and there was no information about the normalization of these samples and there wasn't a zero time point available for post normalization.

**Significant genes:** Genes reported as environmental stress genes were considered to contain the set of differentially expressed genes from Gasch et al.

## Causton et al

Causton HC, Ren B, Koh SS, Harbison CT, Kanin E, Jennings EG, Lee TI, True HL, Lander ES, Young RA: **Remodeling of yeast genome expression in response to environmental changes**. *Mol Biol Cell* 2001, **12**(2):323-337.

**Retrieving data:** Normalized data (presumably **non-log intesities**) and Affymetrix prensense/absense calls were provided in a single file at: <http://younglab.wi.mit.edu/cgi-bin/young/ov.cgi?s=10&d=env&dd=he>. In the article they have further processed the data by taking fold changes against the zero time point of time series.

**Post normalization**: The data was quantile normalized to make the samples from different arrays to have similar scale and means. After this the data was converted to ratios by dividing by the zero time point of each series.

**Significant genes:** Genes fold change larger than 3 (log2 ratio cutoff of ± log_2_(3)=1.58) were defined to be the significant genes from Causton et al.

# Acid tolerance and/or acid production data sets

## Kawahata et al

Kawahata M, Masaki K, Fujii T, Iefuji H: **Yeast genes involved in response to lactic acid and acetic acid: acidic conditions caused by the organic acids in Saccharomyces cerevisiae cultures induce expression of intracellular metal metabolism genes regulated by Aft1p**. *FEMS Yeast Res* 2006, **6**(6):924-936.

**Retrieving data:** Full gene expression data was not available, only supplement tables listing up- or down regulated genes for each comparison. The lists were extracted from Excel and the reported gene expression ratios were combined to form a matrix with 6 columns ( = number of comparisons) and 227 rows ( = number of genes reported as changed in at least one comparison) .

**Post normalization**: The ratios were converted to log2 ratios.

**Significant genes:** The paper supplement only listed the genes up- or down- regulated in each comparison in the study. These genes were taken as the significant genes from Kawahata et al.

## De Nobel et al

de Nobel H, Lawrie L, Brul S, Klis F, Davis M, Alloush H, Coote P: **Parallel and comparative analysis of the proteome and transcriptome of sorbic acid-stressed Saccharomyces cerevisiae**. *Yeast* 2001, **18**(15):1413-1428.

**Retrieving data:** Full gene expression data was not available, only tables listing up- or down regulated genes. The data was copy-pasted from the pdf document and saved in textual format. The expression data contained ratios.

**Post normalization**: The ratios were converted to log2 ratios.

**Significant genes:** The paper only listed the up- or down- regulated genes. These genes were taken as the significant genes from de Nobel et al.

## Ro et al

Ro DK, Ouellet M, Paradise EM, Burd H, Eng D, Paddon CJ, Newman JD, Keasling JD: **Induction of multiple pleiotropic drug resistance genes in yeast engineered to produce an increased level of anti-malarial drug precursor, artemisinic acid**. *BMC Biotechnol* 2008, **8**:83.

**Retrieving data:** Full gene expression data was not available, only supplement tables listing up- or down regulated genes. Log2ratios between aremisinic acid producing and control strain were available for about 2000 genes. In the provided data negative numbers mean that the expression is higher in the artemisinic acid producing strain than in the strain making the precursor.

**Post normalization**: For the purpose of combining this data with the rest of the data the log2ratio is inverted. So in any figures made with this data numbers above one mean higher expression in the artemisinic acid producing strain.

**Significant genes:** The paper supplement separately listed genes that were more than 2 fold up- or down- regulated in the study. This set of genes was taken as the significant genes from Ro et al.

## Hirasawa et al

Hirasawa T, Ookubo A, Yoshikawa K, Nagahisa K, Furusawa C, Sawai H, Shimizu H: **Investigating the effectiveness of DNA microarray analysis for identifying the genes involved in l-lactate production by Saccharomyces cerevisiae**. *Appl Microbiol Biotechnol* 2009, **84**(6):1149-1159.

**Retrieving data:** The gene expression data was provided in pdf-documents listing the gene fold changes for each of the two comparisons (two replicates each). The data was copy-pasted from the pdf documents and saved in textual format. The expression data contained ratios.

**Post normalization**: The ratios from replicate measurements were averaged and the data was converted to log2 ratios.

**Significant genes:** The paper supplement separately listed genes that were more than 2 fold up- or down- regulated in the study. This set of genes was taken as the significant genes from Hirasawa et al.

## Mira et al

Mira NP, Becker JD, Sa-Correia I: **Genomic Expression Program Involving the Haa1p-Regulon in Saccharomyces cerevisiae Response to Acetic Acid**. *Omics* 2010, **14**(5):587-601.

**Retrieving data:** The raw data was available as CEL-files at <http://www.ebi.ac.uk/arrayexpress/experiments/E-MEXP-2740>. The data was downloaded using the ArrayExpress package in R/Bioconductor. The raw data was normalized using the rma function from the “affy” package in R/Bioconductor.

**Post normalization**: The normalized expression data was transformed to log2ratios using the “limma” package in R/Bioconductor. Log2ratios were computed between the acid tolerance condition and the control condition for each strain (haa1 deletion strain, wild type strain).

**Significant genes:** The significance tests were conducted using the “limma” package in R/Bioconductor. The fold change cutoff was set to 1.5 (log2ratio cutoff of ±0.584963). P-value threshold was 0.01. The significance test were conducted without correction for multiple testing.

## Schuller et al

Schuller C, Mamnun YM, Mollapour M, Krapf G, Schuster M, Bauer BE, Piper PW, Kuchler K: **Global phenotypic analysis and transcriptional profiling defines the weak acid stress response regulon in Saccharomyces cerevisiae**. *Mol Biol Cell* 2004, **15**(2):706-720.

**Retrieving data:** Data was available in Excel format at <http://www.at.embnet.org/molg/kuchler/repository/>. Based on the article text and the nature of the data in the spread sheet it was concluded that the data contains **ratios (non-logaritmic)** from the two color microarray. The data has been normalised so that the mean/median ratio across the whole array is 1. There are several (6-8) replicates for each measurement. The data contained quite a lot of missing values.

**Post normalization**: The ratios from replicate measurements were averaged and the data was converted to log2 ratios.

**Significant genes:** Genes with log2ratio larger than 1 or smaller than -1 were defined to be the significant genes from Schuller et al.

## Abbott et al

Abbott DA, Knijnenburg TA, de Poorter LM, Reinders MJ, Pronk JT, van Maris AJ: **Generic and specific transcriptional responses to different weak organic acids in anaerobic chemostat cultures of Saccharomyces cerevisiae**. *FEMS Yeast Res* 2007, **7**(6):819-833.

**Retrieving data:** The raw data was available as CEL-files and as GSE5926_family.soft.gz –file at <http://www.ncbi.nlm.nih.gov/geo/query/acc.cgi?acc=GSE5926> .The data was downloaded using the ArrayExpress package in R/Bioconductor. There was a problem with one of the arrays, GSM137686.CEL, and this was discarded from further analysis. The raw data was normalized using the rma function from the “affy” package in R/Bioconductor.

**Post normalization**: The normalized expression data was transformed to log2ratios using the “limma” package in R/Bioconductor. Log2ratios were computed between the acid tolerance condition and the control condition.

**Significant genes:** The significance tests were conducted using the “limma” package in R/Bioconductor. P-value threshold was 0.005, and the FDR [Benjamini and Hochberg, 1995] method was used for adjusting the p-values for the purpose of correcting for multiple testing. The significance tests were conducted without fold change cutoff.

# References

Benjamini, Y., and Hochberg, Y. (1995). Controlling the false discovery rate: a practical and powerful approach to multiple testing. Journal of the Royal Statistical Society Series B, 57, 289-300.
